# Importance of categories of crime for predicting future violent crime among handgun purchasers in California

**DOI:** 10.1186/s40621-023-00462-5

**Published:** 2023-11-09

**Authors:** Aaron B. Shev, Mona A. Wright, Rose M. C. Kagawa, Garen J. Wintemute

**Affiliations:** Violence Prevention Research Program, Department of Emergency Medicine, University of California, Davis, Sacramento, CA USA

**Keywords:** Firearm, Gun, Violence, Criminal history, Violent crime

## Abstract

**Background:**

Prohibiting the purchase and possession of firearms by those at risk of violence is an established approach to preventing firearm violence. Prior studies of legal purchasers have focused on convictions for specific crimes, such as violent misdemeanors and driving under the influence (DUI). We broaden that line of inquiry by investigating and comparing the associations between prior arrests for most categories of crime and subsequent arrest for violent offenses among legal handgun purchasers in California.

**Methods:**

In this longitudinal cohort study of 79,678 legal handgun purchasers in California in 2001, we group arrest charges prior to their first purchases in 2001 according to categories defined by the Uniform Crime Report (UCR) Handbook. We use a gradient boosting machine to identify categories of offenses that are most important for predicting arrest for violent crime following firearm purchase. For each category identified, we then estimate the difference in risk of subsequent arrest for a violent offense using survival regression models.

**Results:**

We identified eight crime categories with high predictive importance: simple assaults, aggravated assaults, vehicle violations, weapon, other crimes, theft, drug abuse, and DUI. Compared to purchasers with no prior arrests, those with a prior arrest for any one of the eight important categories and no other categories were found to be at increased risk of arrest for a Crime Index-listed violent crime (murder, rape, robbery, aggravated assault), with the greatest estimated risk corresponding to the simple assault UCR category (adjusted hazard ratio 4.0; 95% CI 2.8–5.9). Simple assault was also associated with the greatest risk for subsequent arrest for firearm violence (adjusted hazard ratio 4.6; 95% CI 2.4–9.0) and any violent offense (adjusted hazard ratio 3.7; 95% CI 2.7–5.0).

**Conclusion:**

The findings of this study suggest that prior arrests for a broad array of crimes, both violent and non-violent, are associated with risk of subsequent violent crimes, including Crime Index-listed violent crimes and firearm violence, among legal purchasers of firearms. Current policies aimed at restricting access to firearms for individuals at increased risk of violence should be re-examined considering these findings.

**Supplementary Information:**

The online version contains supplementary material available at 10.1186/s40621-023-00462-5.

## Introduction

There were 47,286 deaths from firearm violence in the USA in 2021—20,958 homicides and 26,328 suicides (Centers for Disease Control and Prevention 2023)—and an estimated 326,894 violent victimizations (robbery, rape, aggravated assault) involving firearms (Bureau of Justice Statistics 2023). In the 10 years from 2012 to 2021, the number of American civilians dying from firearm violence exceeded that of American combat deaths in World War II (DeBruyne 2018).

One widespread approach to preventing firearm violence is to prohibit purchase and possession of firearms by persons believed to be at increased risk for violence. Federal law establishes such prohibitions for persons convicted of felonies and domestic violence misdemeanors, subject to certain restraining orders related to violent behavior, and others (18 USC § 922(d)).

Individuals with non-prohibiting criminal histories remain able to purchase firearms. They account for 17% of legal firearm purchasers in California (the only estimate available at present) and likely a greater percentage in states where there are fewer criminal history prohibitions (Pear et al. 2021). Compared to firearm purchasers with no prior criminal histories, purchasers with a criminal history are at substantially increased risk for future violence, including homicide, rape, robbery, aggravated assault, and intimate partner violence. Persons convicted of violent misdemeanors were found to have seven times the risk of arrest for a future offense compared to those with no prior criminal history (Wintemute et al. 1998). Separately, those convicted of intimate partner violence (Tomsich et al. 2022) have been found to be at 3 times the risk of subsequent arrest for a violent crime compared to those with no criminal history.

It has long been known that a prior history of non-violent crime also predicts future violence. Substance use offenses are an example. Firearm purchasers who were previously convicted for driving under the influence (DUI) have been shown to be at 2 to 3 times the risk for an arrest for a violent crime (Wintemute et al. 2018; Kagawa et al. 2020; Laqueur et al. 2019). This evidence notwithstanding, in most states, persons convicted of misdemeanor crimes of violence (excepting intimate partner violence) and DUI crimes are not prohibited from purchasing firearms. Drug offenses (Pallin et al. 2022) have been established as risk factors as well. More generally, 2 studies have found a roughly fivefold increase in risk for future violence among firearm purchasers associated with convictions for non-violent misdemeanors generally, but they did not differentiate among the many categories of non-violent misdemeanors (Wintemute et al. 1998; Wright and Wintemute 2010). Absent such differentiation, policymakers lack the specific evidence needed to accurately identify those who are at increased risk of violence in the future. In complementary work, Piquero et al. recent review (Piquero et al. 2012) of longitudinal studies, found that violent offenders were characterized as being frequent non-violent offenders prior to their violent offense. The studies reviewed by Piquero et al. (2012) did not focus on firearm owners, however.

This study is designed to address the gaps in the current evidence base, providing specific information on risk for future violence among firearm purchasers associated with specific categories of crime and allowing for direct comparison of the statistical importance of each category. We examine the association between prior arrests at the time of purchase, for crimes across nearly the entire spectrum of criminal activity, and risk for future violence among legal purchasers of handguns in California. We take a data-driven approach that considers multiple crime types simultaneously to identify which are most statistically important. To our knowledge this is the first study to take such a multifaceted approach to this population of legal handgun purchasers. In doing so, it provides for the first time the evidence that should underlie a broad consideration of the question, among prospective purchasers of firearms, who is at unacceptably high risk for future violence?

## Methods

This study builds on the work of Kagawa et al. (2020) by providing a broad look at the association between criminal histories and subsequent arrest for violent crime. We use a records-based longitudinal cohort design that enrolls a large population of legal purchasers of handguns. Data is assembled from multiple sources on details of prior criminal history and potential risk factors for our prespecified violent crime arrest outcomes that existed at the time of enrollment. The population is followed using record surveillance until we can no longer verify residence in California, death, or the period of observation ends. The design is described in detail in our study protocol (Wintemute et al. 2016).

### Study population

Our cohort comprises all legal handgun purchasers in California who purchased a handgun in 2001 and were age 21–49. We refer to the first handgun purchase in 2001 as the index purchase although purchasers may have purchased handguns in previous years. Purchasers enter the cohort beginning 10 days following the index purchase to account for California’s 10-day waiting period. Bounding the ages at index purchase from 21 to 49 captures purchasers from the minimum age of purchase at the lower bound to an upper bound where criminal activity is well documented to decrease substantially (Loeber and Farrington 2014). The cohort was identified through the California Dealer’s Record of Sale (DROS) system–a database of all legal firearm transfers in California.

Each study participant was followed through December 31, 2013, or until a prior date when the participant could no longer be confirmed to be living in California using public records. Public records used to verify active residence included the California Death Statistical Master File, California voter registration records, and DROS records. Additionally, if participants were unaccounted for a duration of at least three years, we queried Lexis-Nexis Public Records to identify activity within California.

### Exposures and outcome

The main exposure was a history of at least one arrest for any one of 27 categories of crime prior to the index handgun purchase. The categories were defined by the Federal Bureau of Investigation’s Uniform Crime Reporting (UCR) handbook (U.S. 2004) with an additional category for Vehicle Code violations as defined by the State of California (https://leginfo.legislature.ca.gov/). The California Vehicle Code provides statutes for permitting drivers, registration of vehicles, sale of vehicles, and other administrative matters related to vehicles in addition to vehicle and traffic violations. Moving forward, references to UCR categories will be assumed to be inclusive of the additional vehicle violations category. All crimes were coded into one of the 27 mutually exclusive UCR crime categories based on code and statute, description of the crime, and offense level–misdemeanor or felony. Crimes that could not be categorized on the information present and infraction offenses were not included in the analysis. Individuals with multiple arrest charges could be represented in more than one UCR category.

The primary outcome of interest was an arrest for a violent crime listed in the Crime Index published by the Federal Bureau of Investigation (murder, rape, robbery, and aggravated assault) (Federal Bureau of Investigation 2004). With the limited number of observations in certain UCR categories, it was statistically beneficial to use arrest charges, as opposed to convictions, as an outcome. Though not all arrest charges lead to a conviction, arrests are reported more rapidly and completely than convictions (Bureau of Justice Statistics 2011). As a sensitivity analysis, we consider convictions for Crime Index-listed violent (CIV) crimes as an outcome. Secondary outcomes included arrest charges for any firearm-related violent crimes as well as at least one arrest charge for any violent crime as defined by the Federal Bureau of Investigation and the World Health Organization (Federal Bureau of Investigation 2004, 2019; Definition and typology of violence 2019), a broad category for any crime that may be described as violent. Simple assaults, for example, are contained in this outcome but are not Crime Index-listed. The firearm-related violent crime outcome is a subset of general violent crime outcome and were identified by statute, offense description, and qualifiers on an offense that indicate a firearm involvement. Additional File 1 details all violent offenses used in the primary and secondary outcomes.

### Covariates

We controlled for both individual-level and community-level characteristics in our analysis. Purchaser gender, and age, as reported in the Dealer’s Record of Sale, are included in all models. Age and gender have well-established relationships with crime risk (Shulman et al. 2013). Community characteristics have also been shown to be informative predictors of risk (Goin et al. 2018). Using the address associated with the index handgun purchase, we controlled for census-tract demographics from the American Community Survey: population size; the proportion of people ages 20–24 among the population ages 20–44; and the percentages of the population that are male. We used an index for socioeconomic status produced from a linear combination of standard education, wealth, and employment indicators (see Additional File 2 for details on this index). A variable for alcohol outlets per square mile in each census tract, shown to be associated with violent crime (Trangenstein et al. 2018), was created using counts of four types of alcohol licenses (bar/pub/tavern, restaurant beer wine, restaurant spirits, and off-premise) (California Department of Alcoholic Beverage Control 2019). Finally, our models controlled for county violent crime, property crime (Uniform Crime Reporting Program 2017), and approximate firearm suicide rates. The latter is approximated with the commonly used ratio–the proportion of firearm suicides out of total suicides by county (Azrael et al. 2004; Based on data received by the authors from California Department of Public Health 2015).

Our previous work found that the inclusion of time-varying covariates to account for movement of subjects to residences in different communities and changes within a community did not substantively change estimates (Wintemute et al. 2018). As such, we only use values for community characteristic variables recorded at baseline in this analysis. Unadjusted survival models are also fit as a sensitivity analysis.

### Statistical approach

The analysis was carried out in two stages. In the first stage, we performed a variable importance analysis, a method for comparing covariates by measuring the improvements in a model’s predictive performance from the inclusion of each covariate. We then identify the prior arrest UCR exposure categories with the highest relative values of the importance metric for predicting a future violent crime arrest. For the second stage, we fit survival regression models to estimate adjusted hazard ratios (AHR) for each UCR category determined to be important. This design provides a data-driven approach to test only relevant exposures and control the number of hypothesis tests conducted.

For the variable importance analysis, we used a gradient boosted machine (GBM) with Cox Proportional Hazards loss functions to predict the time-to-event for a given outcome from prior arrest for a UCR category. The GBM provides a nonparametric method to simultaneously consider all 27 levels of our exposure and identify only the criminal histories that are most predictive of our outcome (Friedman 2001). An interaction depth of three was chosen to allow interactive effects to be considered. The number of trees was determined using tenfold cross validation. All covariates described above were used in the variable importance analysis in addition to indicator variables for an arrest charge or conviction prior to index purchase for each of the 27 UCR categories. Importance was measured using relative influence (Friedman 2001). The top UCR categories were chosen by clustering crime categories by absolute distance in relative influence values using hierarchical clustering with Ward’s method to minimize within cluster variance (Ward 1963). In practice, this will separate clusters when variable importance decreases by a relatively large amount. We choose a cut point that results in two clusters, and the cluster of UCR categories with greater relative influence was chosen for the second stage of the analysis.

We fit a mixed effects Cox Proportional Hazards regression model for each UCR category determined to be important for predicting future violence. In each model, the UCR category of interest was parameterized as a four-level variable indicating purchasers with (1) prior arrests for the UCR category of interest and other UCR categories, (2) prior arrests for only the UCR category of interest, (3) prior arrests only for UCR categories other than the one of interest, or (4) no criminal history. Random intercepts were included for census tract nested within county. *P*-values were adjusted using Bonferroni-Holm to simultaneously test the significance of all UCR categories at family-wise significance level of α = 0.05, and we constructed corresponding 95% family-wise confidence intervals for all adjusted hazard ratios for the UCR arrest coefficients. Any purchasers identified to be deceased or who were unable to be confirmed as living in California after three years of inactivity were treated as censored. All models included the covariates described above and were fit using R 4.2.0 (R Core Team 2022) with the gbm (version 1.2.8) (Greenwell et al. 2022) and coxme (version 2.2–18.1) (Therneau 2022) packages.

## Results

### Description of study population

We identified 79,678 legal handgun purchasers for our study population. After excluding purchasers with missing race or community information (*n* = 664), and those with no valid follow-up information or never picked up their first handgun purchase (*n* = 136), 78,878 handgun purchasers remained. During the observation period, 1997 purchasers became censored due to death and another 9105 purchasers moved out of state and became censored.

Of purchasers in the cohort, 91% were male and 69% were white with just under 17% of purchasers having a criminal history (arrest charges or convictions) prior to their index handgun purchase in 2001. The most common UCR categories in prior criminal histories were other crimes, weapon offenses, and theft, with 3.8%, 3.6%, and 3.3% of the cohort having at least one arrest corresponding to those categories, respectively. The “other crimes” UCR category is defined by the UCR program to include violations of laws not specifically identified by the other categories. The most common charges within other crimes among our data were trespassing and presenting false identification to a peace officer or other official. Arson, embezzlement, and gambling were the least common crime categories with less than 0.1% of the cohort having a prior arrest for one of these categories. Just 0.02% of crimes were not able to be identified, and 1.09% of items on criminal histories were omitted as they were administrative or not crimes. Pear and colleagues (Pear et al. 2021) describe this cohort in greater detail.

**Fig. 1:**
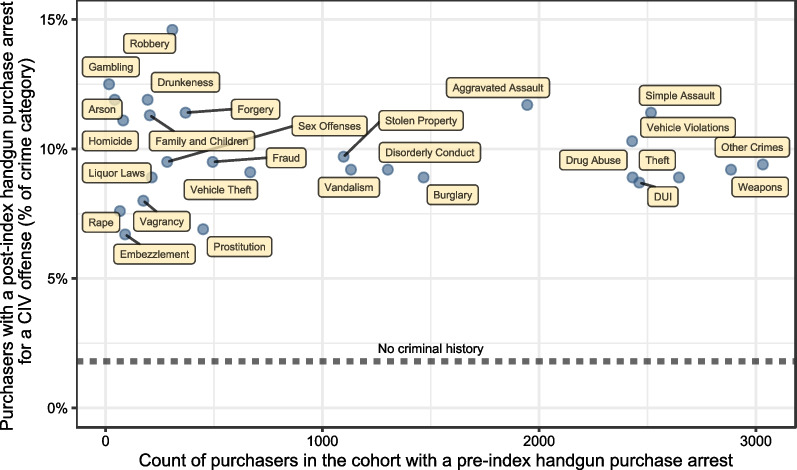
Scatterplot of prior arrest frequency by percent of purchasers for a given category with a subsequent Crime Index-listed violent offense arrest

Among purchasers with a prior criminal history, the percentage of purchasers with at least one arrest for a CIV crime following the index handgun purchase ranged from 6.7% for those with a prior embezzlement arrest to 12.5% for those with a prior gambling arrest. In comparison, those with no prior criminal history had a subsequent CIV arrest incidence of only 2.8%. Figure 1 displays the number of purchasers arrested prior to their index handgun purchase for each UCR category in the cohort by the incidence of CIV crimes within a given UCR category, and for the purpose of describing these data, we identify three distinct groups visually: high frequency of prior arrest with high frequency of subsequent CIV arrest, low frequency prior arrest with high frequency subsequent arrest, and low frequency prior arrest with low frequency subsequent arrest. Among the prior arrest UCR categories with high frequency prior arrest and high frequency subsequent arrest, the purchasers with prior aggravated assault arrests or prior simple assault arrests had the highest percentages of purchasers with subsequent CIV arrests–11.7% and 11.4%, respectively. A complete table of counts of prior and subsequent arrests for all exposures and outcomes is available in Additional File 3.

### Variable importance analysis

**Fig. 2:**
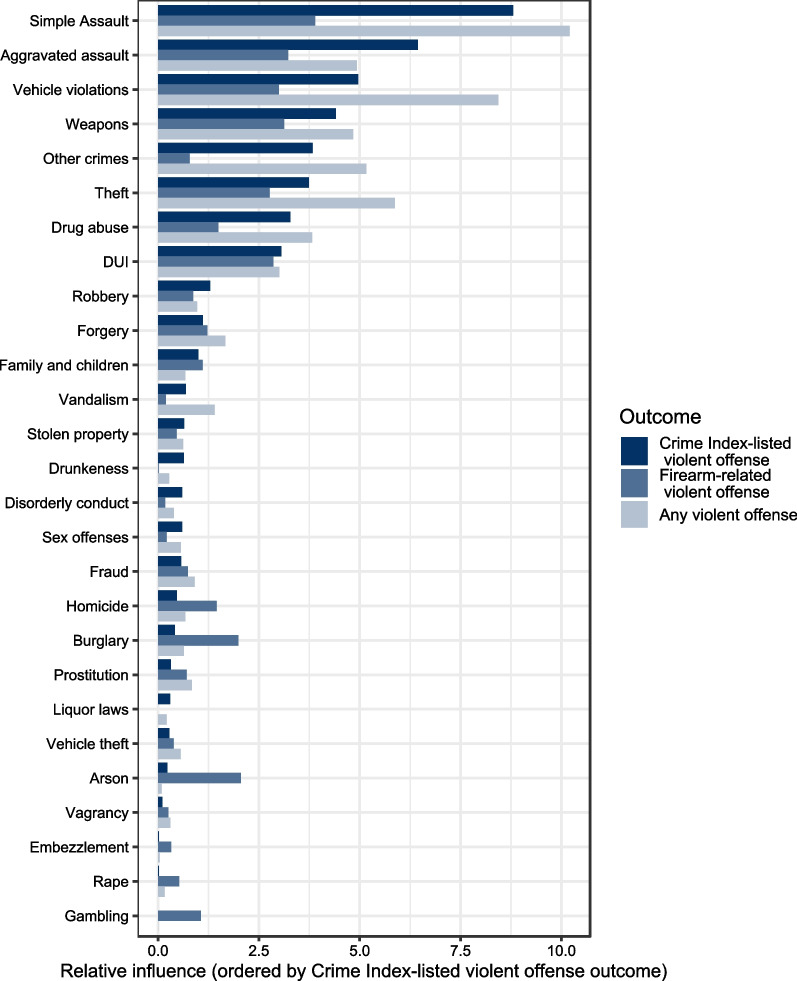
Relative influence statistics for UCR category arrests prior to index firearm purchase for predicting a subsequent arrest for a violent offense

The variable importance analysis for the CIV outcome determined a group of eight UCR categories that corresponded to a relatively greater increase in model fit. These eight categories, in order of decreasing importance, were: simple assault, aggravated assault, vehicle violations, weapons violations, other crimes, theft, drug abuse, and DUI. A large drop in relative influence following DUI provided a natural cut-point, identified by the hierarchical clustering, to partition the categories into high and low importance groups. Figure 2 depicts all categories in order of decreasing relative influence for all outcomes. The same crime categories were found to be important for the any violent crime outcome with some changes in the order, while the firearm-related violent crime outcome dropped drug abuse and other crimes from the top cluster. Clustering outputs for all outcomes are displayed in Additional File 4.

### Survival analysis

**Fig. 3:**
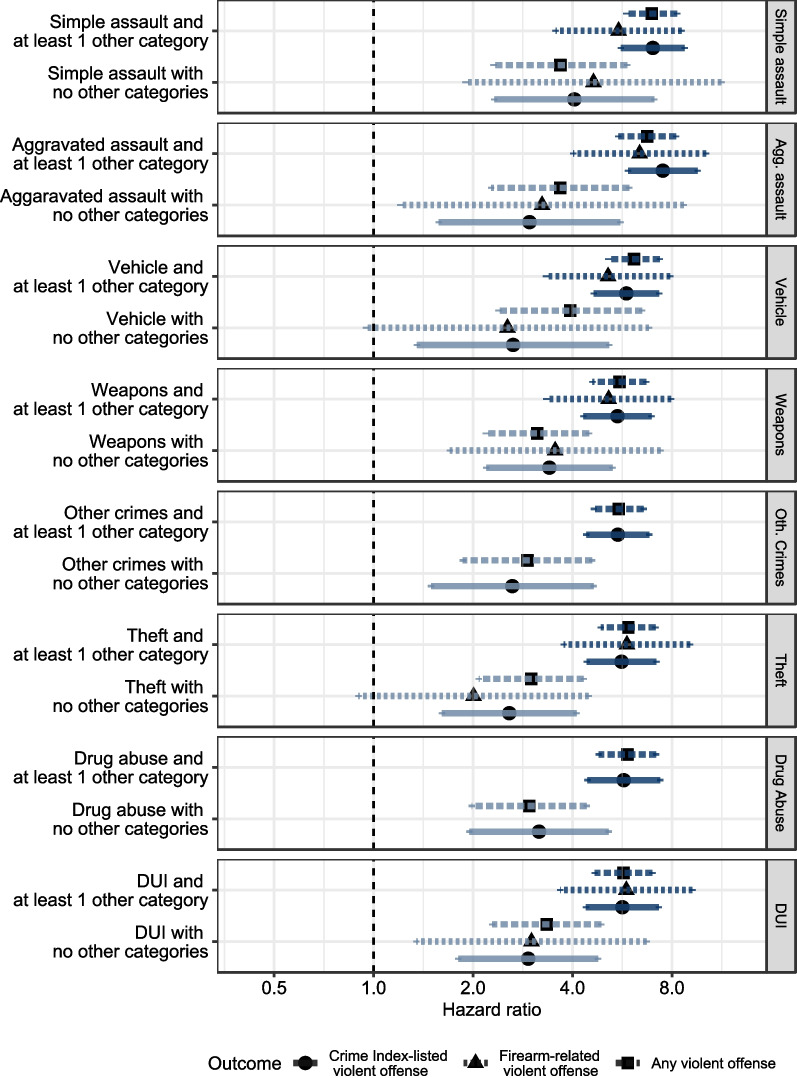
Adjusted hazard ratios and corresponding 95% family-wise confidence intervals for isolated UCR categories and multiple UCR categories

For each of the UCR categories selected as statistically important for a given outcome, we fit a mixed effects Cox proportional hazards regression to estimate the effect of criminal histories on time until subsequent arrest for a CIV crime. All prior arrest UCR categories were associated with an increased risk of subsequent arrest for a CIV crime (statistically significant at a familywise α = 0.05 level). The adjusted hazard ratios for isolated UCR categories ranged from 2.6 (95% CI = 1.8, 3.6) for theft to 4.0 (95% CI = 2.8, 5.9) for simple assault. For multiple UCR categories (i.e., prior arrests for both the category of interest and other UCR categories), the hazard ratios ranged from 5.4 (95% CI = 4.7, 6.3) for weapons violations to 7.5 (95% CI = 6.4, 8.7) for aggravated assault. These results are displayed visually in Fig. 3.

We observed similar associations between arrest history and subsequent arrest for firearm violence and subsequent arrest for any violence, with a few notable differences. The associations between the isolated categories of prior criminal history for vehicle offenses, and theft and subsequent arrest for firearm violence were not statistically significant. Hazard ratios for all prior multiple arrest categories were statistically significant for subsequent firearm violence arrests and arrests for any violent crime. All adjusted hazard ratios for prior arrests with 95% confidence intervals are shown in Fig. 3 and in the table in Additional File 5.

Model coefficients and associated standard errors are given in the tables in Additional File 6. For all models and outcomes, gender, age at index purchase, and census tract SES are statistically significant. All models for the subsequent arrest for a CIV offense outcome estimate about 50% greater risk for men across all exposures, roughly 75% greater risk for a subsequent arrest for firearm violence across all exposures, and roughly 45% greater risk for subsequent arrest for any violent offense across all exposures. Across all outcomes and exposures there was an estimated 3–4% decrease in risk for each additional year of age at index purchase; higher values of census tract SES were also associated with decreasing risk. The number of firearms legally purchased in California prior to the index purchase is statistically significant in some models for the CIV outcome, no models for the firearm violence outcome, and all models for the any violent offense outcome. For the CIV and any violence outcomes, each additional gun owned at the time of the index handgun purchase was associated with approximately 1% lower risk of arrest.

### Sensitivity analysis

Sensitivity analyses showed similar results as compared to the main analysis. Cox regressions for the eight categories selected for the CIV crime outcome were refit using subsequent conviction for a CIV crime instead of an arrest. The variable importance analysis found the same eight categories to be the most important with some minor differences in ordering. All hypothesis tests reached the same conclusions apart from the effects for the category of only “other crimes” in isolation and only DUI in isolation. All results for this analysis may be found in Additional File 7. Unadjusted results were obtained for all survival models. All results aligned with the main analysis with the exception that theft became statistically significant for arrest for firearm-related violent crime. These results are shown in Additional File 8.

## Discussion

To our knowledge, this is the first study to look both broadly and in detail at associations between prior criminal history and subsequent violent criminal activity among legal purchasers of firearms. From 27 categories of crimes, we identified a group of eight crime categories to have the greatest importance in prediction of subsequent arrest. Each of the eight categories was associated with an elevated risk of subsequent arrest for a Crime Index-listed violent crime, with adjusted hazard ratio estimates ranging from 2.4 to 3.6. Adjusted hazard ratio estimates nearly doubled in magnitude when arrests for multiple categories were present in a purchaser’s criminal history suggesting the presence of a dose–response relationship for the number of arrests. In concordance with prior research (Piquero et al. 2012), arrests for non-violent crimes as well as arrests for violent crimes were associated with increased risk for future violence; note that of the 8 most important categories we identified, 5 did not involve violence or weapons (Fig. 2). In addition, the eight categories found to be the most important were also the most common classes of criminal histories observed.

Taken together, these findings suggest policies seeking to prevent firearm violence by prohibiting purchase and possession of firearms by high-risk individuals should be re-examined with an eye to broadening the prohibition criteria. Current evidence suggests that expanded restrictions would be effective at the individual level (Wintemute et al. 2001). At an individual level, policies that remove firearms from high-risk individuals remove the potential for a firearm to be involved in a future crime. The effect of an intervention may also not be limited to the type of crime that triggered the intervention, so there may be a diffusion of benefit to non-firearm related violent crime by removing a firearm (Guerette and Bowers 2009). Effects at the population level would be a function of individual-level effects and the number of persons affected by changes in policy; they would likely be greatest in states with the fewest restrictions in place at the time of the policy change.

In a previous study on a 1977 cohort of legal firearm purchasers in California found handgun purchasers with a prior misdemeanor conviction had 5.1 times the risk of being charged with an offense post-purchase as compared with handgun purchasers that had no prior criminal record (Wintemute et al. 1998). The risk observed in the present cohort is lower, likely at least in part, because many convictions for violent misdemeanors would now be prohibiting in California, and the remaining purchasers with misdemeanor convictions may have a lower risk profile. The findings of this study also expand substantially on our prior work, which found increases in risk for future violence associated with convictions for intimate partner violence (Tomsich et al. 2022) and DUI (Kagawa et al. 2020).

Somewhat unintuitively, prior arrest for simple assault, and not aggravated assault, had the largest estimated risk. This may be a result of California purchasing laws that prohibit those with a violent misdemeanor conviction from purchasing firearms in California. Those with a prior aggravated assault arrest who were subsequently able to purchase a firearm were able to do so either because they were not convicted or convicted of a different non-violent charge not leading to a prohibition or because they had a prohibition that had expired. These purchasers are convicted at a lower rate–just 6% as opposed to 19.8% for simple assault–and therefore may represent a lower risk group than expected. For all three outcomes, either vehicle offenses or DUI was estimated to be one of the top three most important prior arrest categories, but not both. Moreover, for the CIV outcome, DUI was ranked 8th in relative influence while estimated to have a larger single category hazard ratio. As many as 75% of DUI offenders with a suspended license report driving while the license is suspended–the most common vehicle violation in these data–creating potential overlap between the categories (Ross and Gonzales 1988). This is reflected in the data, as we observe a high correlation between having at least one arrest for a vehicle offense and at least one arrest for a DUI among individuals with a criminal record explains this discrepancy. These results agree with our previous work measuring the association between DUI conviction and subsequent violent crime (Wintemute et al. 2018).

As a consequence of the relative influence statistic’s construction, the variable importance analysis favors selecting crime categories that have both a large magnitude of association with the outcome and are more prevalent in the population. Indeed, Fig. 1 shows the top-eight most important categories of prior crimes noticeably separated from all other categories, so it is no surprise that we found such a distinct cluster in the relative importance statistic. However, the remaining categories should not necessarily be interpreted to have low risk. There were other categories with high individual risk, but low population prevalence. For example, just under 12% of purchasers with a prior arrest for an arson crime were later arrested for a CIV crime, but only 42 purchasers in the cohort had a prior arrest for an arson crime.

Our study is subject to some limitations. The generalizability of the results is limited geographically by only following handgun purchasers in California as well as temporally by restricting our cohort to those who legally purchased a handgun in 2001. Second, our study relies on the use of criminal records. Arrests are an imperfect measure of actual criminal activity as much criminal activity does not lead to an arrest and some arrests do not necessarily indicate any criminal activity occurred. Arrests are also subject to biases in policing and may overestimate or underestimate risk for some people as a result.

## Conclusion

We categorized the criminal histories of legal handgun purchasers in this longitudinal cohort study and identified eight categories of prior crimes that were most important in prediction of subsequent violent crimes. Purchasers with prior offenses in any of the eight categories identified were found to be at significantly increased risk for subsequent arrest for a Crime Index-listed violent crime. While careful review of the equitability of associated criminal justice system outcomes is needed, the findings presented here suggest additional criteria not previously considered for policies that prohibit purchase and possession of firearms by high-risk individuals would be effective in preventing firearm violence. Considering this, such policies should be re-examined to consider broadening the scope of prohibitions.

### Supplementary Information

**Additional file 1.** Offenses categorized as violent–a table of all offenses categorized as violent with indicators for outcomes in which they were included.

**Additional file 2.** Socioeconomic index description–a description of the components and the construction of the census tract level socioeconomic index used in the modeling.

**Additional file 3.** Crime category frequency table -- frequencies of legal handgun purchasers in the cohort by specified criminal history and outcome.

**Additional file 4.** Hierarchical clustering of crime categories by relative importance–dendrograms of crime categories clustered by relative importance for arrest for a Crime Index-listed violent offense, arrest for a firearm-related violent offense, and arrest for any violent offense.

**Additional file 5.** Tables of survival regression model coefficients–tables of regression coefficients for the adjusted Cox proportional hazards models for the primary and secondary outcomes.

**Additional file 6.** Estimated adjusted hazard ratios and corresponding family-wise 95% confidence intervals table–estimates and Bonferroni-adjusted confidence interval values corresponding to those shown in Figure 3.

**Additional file 7.** CIV conviction sensitivity analysis results table–estimated adjusted hazard ratios and Bonferroni-adjusted family-wise 95% confidence intervals for conviction for a CIV offense.

**Additional file 8.** Unadjusted model sensitivity analysis results table–estimated unadjusted hazard ratios and corresponding family-wise 95% confidence interval values for all outcomes and exposures.

## Data Availability

Restrictions apply to the availability of the data used in this study. The data that support the findings of this study are available to researchers from the California Department of Justice upon request. https://oag.ca.gov/research-center/request-process

## Abbreviations

DUI: Driving under the influence
CIV: Crime Index-listed violence
UCR: Uniform Crime Reporting
