# Supplementary material for: Importance of categories of crime for predicting future violent crime among handgun purchasers in California
Source: Inj Epidemiol. 2023 Nov 9;10:57. doi: 10.1186/s40621-023-00462-5 (PMC10634023; doi:10.1186/s40621-023-00462-5)
Supplement: Supplementary file 1 — Additional file 1. Offenses categorized as violent–a table of all offenses categorized as violent with indicators for outcomes in which they were included. [file 40621_2023_462_MOESM1_ESM.docx]

Additional Files 1. Offenses categorized as violent

| CODE | SECTION | SUB-SECTION(S) | DESCRIPTION | UCR | CRIME INDEX VIOLENCE | FIREARM-RELATED VIOLENCE |
| --- | --- | --- | --- | --- | --- | --- |
| P | 187 | (a) | First degree murder | 1 | x | § |
| P | 192 | (a) | Voluntary manslaughter | 1 | x |  |
| P | 261 | (a), (a)(1), (a)(2), (a)(3), (a)(4) | Rape by force or threat | 2 | x |  |
| P | 262 | (a) | Rape of spouse by force/threat/fear | 2 | x |  |
| P | 264.1 |  | Rape/etc. in concert with force/violence | 2 | x |  |
| P | 211^§^ |  | Robbery | 3 | x | § |
| P | 212.5 | (a), (b), (c)^§^ | Robbery of inhabited building, vehicle, ATM | 3 | x | § |
| P | 213.5 | (a)(1)(A), (a)(2) | Robbery of inhabited dwelling, in concert with others | 3 | x |  |
| P | 215 | (a)^§^ | Carjacking | 3 | x | § |
| P | 148 | (b) | Remove weapon from public officer - not firearm | 4 | x |  |
| P | 149 |  | Assault by public officer | 4 | x |  |
| P | 203 |  | Mayhem | 4 | x |  |
| P | 217 |  | Assault w/intent to kill | 4 | x |  |
| P | 220 |  | Assault with intent to commit rape | 4 | x |  |
| P | 221 |  | Assault to commit other felony | 4 | x |  |
| P | 240 |  | Assault | 4 | x |  |
| P | 241 | (a), (b), (c) | Assault peace officer | 4 | x |  |
| P | 241.1 |  | Assault on custodial officer | 4 | x |  |
| P | 241/243 |  | Assault and battery on peace officer /emergency personal | 4 | x |  |
| P | 243 | (a), (b), (c)(1), (c)(2), (d) | Battery on peace officer/emergency personal w/injury | 4 | x |  |
| P | 243.1 |  | Battery on custodial officer | 4 | x |  |
| P | 243.4 | (a), (b), (c), (d), (e)(1) | Sexual battery | 4 | x |  |
| P | 243.6 |  | Assault or battery on process server | 4 | x |  |
| P | 244 |  | Assault with caustic chemical/etc | 4 | x |  |
| P | 245 | (a), (a)(1), (a)(2), (a)(4), (b), (c) | Assault with deadly weapon or force: possible great bodily injury: not firearm | 4 | x |  |
| P | 245 | (a)(1), (a)(2), (a)(3), (a)(4), (b), (c), (d)(1), (d)(2), (d)(3) | Assault with deadly weapon: firearm | 4 | x | x |
| P | 246 |  | Shoot at inhabited dwelling/vehicle/etc. | 4 | x | x |
| P | 246.3 | (a), (b) | Willful discharge of firearm with gross negligence | 4 | x | x |
| P | 269(A)(1) | (a)(1), (a)(4) | Aggravated sexual assault of child/minor | 4 | x |  |
| P | 273.5 | (a) | Inflict corporal injury: spouse/cohabitee/date | 4 | x |  |
| P | 273.55 |  | Inflict corporal injury: spouse/cohabitee/date: special circumstances | 4 | x |  |
| P | 273a | (1), (a), (a)(1) | Willful cruelty to child: possible injury/death | 4 | x |  |
| P | 273d | (a) | Inflict injury upon child | 4 | x |  |
| P | 368 | (a), (a)(1),  (b)(1), (c) | Cruelty of elderly/dependent adult with great bodily injury/death or mental suffering | 4 | x |  |
| P | 405a |  | Lynching | 4 | x |  |
| P | 417 | (b), (c) | Exhibit firearm in presence peace officer | 4 | x | x |
| P | 417.1 |  | Exhibit firearm in presence of reserve police officer | 4 | x | x |
| P | 417.3 |  | Occupant of motor vehicle exhibit/draw firearm | 4 | x | x |
| P | 422 | (a)^§^ | Threaten crime with intent to terrorize | 4 | x | § |
| P | 422.7 | (a) | Violate civil rights by force/treat | 4 | x |  |
| P | 4501 |  | Assault by prisoner | 4 | x |  |
| P | 4501.5 |  | Battery by prisoner | 4 | x |  |
| P | 664/187 | (a) | Attempted murder | 4 | x |  |
| P | 69 |  | Obstruct/resist executive officer | 4 | x |  |
| P | 76 | (a) | Threaten/etc. elected official/judge/etc. | 4 | x |  |
| V | 23110 | (b) | Throw substance at vehicle with intent of great bodily injury | 4 | x |  |
| P | 136 | (b) | Prevent/dissuade witness/victim by force/etc. | 9 |  |  |
| P | 136.1 | (a)(2), (b)(1), (b)(2), (b)(3), (c), (c)(1) | Prevent/dissuade witness/victim by force/etc. | 9 |  |  |
| P | 140 | (a) | Threaten witness/victim of crime | 9 |  |  |
| P | 148 | (a), (a)(1) | Obstruct/resist public officer | 9 |  |  |
| P | 148.2.1 |  | Interfere with/etc. fireman/rescuer | 9 |  |  |
| P | 148.2.2 |  | Disobey order of fireman/public officer | 9 |  |  |
| P | 240^ǂ^ |  | Assault | 9 |  |  |
| P | 240/242 |  | Assault & battery | 9 |  |  |
| P | 241 | (a) ^ǂ^, (b) ^ǂ^ | Assault on peace officer/emergency personal | 9 |  |  |
| P | 241.4 |  | Assault school district peace office | 9 |  |  |
| P | 241.6 |  | Assault on school employee | 9 |  |  |
| P | 241/243^ǂ^ |  | Assault and battery on peace officer /emergency personal | 9 |  |  |
| P | 242 |  | Battery | 9 |  |  |
| P | 242/243 | (a), (b) | Battery on peace officer /emergency personal | 9 |  |  |
| P | 243 | (e)(1) | Bat Battery: spouse/ex-spouse/date/etc. | 9 |  |  |
| P | 243.2 | (a) | Battery on person on school property | 9 |  |  |
| P | 243.3 |  | Battery on transportation personnel | 9 |  |  |
| P | 243.4 | (a) ^ǂ^, (d) ^ǂ^, (d)(1) | Sexual battery | 9 |  |  |
| P | 243.5 | (a)(1) | Assault or battery on school property | 9 |  |  |
| P | 243.6^ǂ^ |  | Assault or battery on process server | 9 |  |  |
| P | 244.5 | (b) | Assault with stun gun/taser | 9 |  |  |
| P | 245 | (a)(1) ^ǂ^ | Assault w/deadly weapon: not firearm | 9 |  |  |
| P | 273a | (b) | Willful cruelty to child | 9 |  |  |
| P | 273d | (a) ^ǂ^ | Inflict injury/etc. upon child | 9 |  |  |
| P | 368 | (b), (c) ^ǂ^ | Cruelty to dependent adult | 9 |  |  |
| P | 69^ǂ^ |  | Obstruct/resist executive officer | 9 |  |  |
| P | 71 |  | Threaten school/public officer/employee | 9 |  |  |
| U | 18 113 | (e) | Simple assault | 9 |  |  |
| P | 146a |  | Impersonate public officer/etc. | 11 |  |  |
| P | 12024 |  | Possess deadly weapon: commit assault | 15 |  |  |
| P | 12034 | (b) | Discharge firearm from vehicle | 15 |  | x |
| P | 12303.2 |  | Possess explosive/etc. device in public | 15 |  |  |
| P | 12303.3 |  | Use/etc. explosive/etc. device: intent to injury | 15 |  |  |
| P | 12355 | (b) | Possess boobytrap device with intent to use | 15 |  |  |
| P | 12403.7 | (a), (a)(5), (a)(7), (a)(8) | Illegal possession/use of tear gas/tear gas weapon | 15 |  |  |
| P | 246.3 | (a), (b) | Willful discharge of firearm with gross negligence | 15 |  | x |
| P | 248 |  | Impair aircraft with light/etc. | 15 |  |  |
| P | 417 | (a), (a)(2) | Exhibit deadly weapon/firearm | 15 |  | x |
| P | 417 | (a)(1) | Brandishing weaponry, not firearm | 15 |  |  |
| P | 417.25 | (a) | Threaten person with laser scope with intention of causing fear | 15 |  |  |
| P | 417.4 |  | Brandishing firearm replica | 15 |  | x |
| P | 467 |  | Possess weapon to commit assault | 15 |  |  |
| P | 286 | (d)(1) | Sodomy in concert with force | 17 |  |  |
| P | 288 | (b)(1) | Lewd and lascivious acts with child under 14 yrs.: with force/etc. | 17 |  |  |
| P | 288.5 | (a) | Continuous sexual abuse of child | 17 |  |  |
| P | 288a | (a), (b), (d) | Oral copulation | 17 |  |  |
| P | 289 | (a), (a)(1), (b) | Sexual penetration with foreign object/etc. with force/etc. | 17 |  |  |
| P | 273a | (2) | Willful cruelty to child | 20 |  |  |
| P | 277 |  | Deprive custody right of another | 20 |  |  |
| P | 415 | (1), (2), (3) | Fight/challenge fight/ unreasonable noise/offensive words in public place | 24 |  |  |
| P | 415.5 | (a)(1) | Fight/challenge fight on university/etc. | 24 |  |  |
| U | 36 2.34 | (a)(1) | Disorderly conduct: fight/etc. | 24 |  |  |
| H | 12680 |  | Discharge fireworks: likely to injure | 26 |  |  |
| L | 6425 |  | Cause employee death/impairment | 26 |  |  |
| P | 136 |  | Prevent/dissuade witness/victim | 26 |  |  |
| P | 136.1 | (a), (a)(2), (b), (b)(1), (b)(2) | Prevent or dissuade witness from giving testimony | 26 |  |  |
| P | 146 | (a) | Make arrest/etc. without authority | 26 |  |  |
| P | 147 |  | Inhumanity to prisoners | 26 |  |  |
| P | 148.1 | (a), (b), (c), (d) | False bomb report | 26 |  |  |
| P | 148.3 | (a) | False report of emergency | 26 |  |  |
| P | 207 | (a), (b) | Kidnapping | 26 |  |  |
| P | 209 | (a), (b), (b)(1) | Kidnapping for ransom or to commit robbery/rape/etc. | 26 |  |  |
| P | 236^§^ |  | False imprisonment | 26 |  | § |
| P | 237 | (a) | False imprisonment with violence | 26 |  |  |
| P | 278 |  | Child stealing | 26 |  |  |
| P | 375 | (a) | Offensive/etc. matter in public place | 26 |  |  |
| P | 404.6 | (a) | Urge riot or destroy property | 26 |  |  |
| P | 405 |  | Riot | 26 |  |  |
| P | 422 | (a) ^ǂ^ | Threaten crime with intent to terrorize | 26 |  |  |
| P | 422.6 | (a) | Violate civil rights by force/threat | 26 |  |  |
| P | 4532 | (a), (b) | Escape jail/etc. with force/violence | 26 |  |  |
| P | 518 |  | Extortion | 26 |  |  |
| P | 519 |  | Extortion by threat | 26 |  |  |
| P | 520 |  | Extortion of property | 26 |  |  |
| P | 601 | (a)(1) | Trespass on residence: execute threat | 26 |  |  |
| V | 23110 | (a) | Throw substance at vehicle | V^€^ |  |  |
| V | 2800.2 |  | Evade peace officer in vehicle disregarding safety | V^€^ |  |  |
| V | 2800.3 |  | Evade peace officer: cause great bodily injury/death | V^€^ |  |  |

Table A2.1 All offenses categorized as violent with indicators for inclusion in the Crime Index-listed primary outcome, and the firearm-related violence outcome. All offenses listed in the table were included in the any violence outcome.

^a^ Code: H = California Health and Safety code; L = California Labor code; P = California Penal code; U = Code of Federal Regulations; V = California Vehicle code

§ Categorized as gun offense if a firearm was used in the commission of the crime

ǂ Categorized as Crime-index violence if offense is charged at the felony level

€ Not a UCR code; categorized as a vehicle code violation
