# Supplementary material for: Importance of categories of crime for predicting future violent crime among handgun purchasers in California
Source: Inj Epidemiol. 2023 Nov 9;10:57. doi: 10.1186/s40621-023-00462-5 (PMC10634023; doi:10.1186/s40621-023-00462-5)
Supplement: Supplementary file 2 — Additional file 2. Socioeconomic index description–a description of the components and the construction of the census tract level socioeconomic index used in the modeling. [file 40621_2023_462_MOESM2_ESM.docx]

Additional files 2. Socioeconomic index

The socioeconomic index captured variation in the percentages of families in poverty, of adults unemployed, of adults with a high school diploma, of adults with a college diploma, and median income at the census tract level in a principal component analysis of the rank transformed data. The index appeared highly reliable (Cronbach’s alpha=0.94, with 80% of the variance explained by the first principal component in 2001, 2005, and 2010 data).
