# Supplementary material for: Importance of categories of crime for predicting future violent crime among handgun purchasers in California
Source: Inj Epidemiol. 2023 Nov 9;10:57. doi: 10.1186/s40621-023-00462-5 (PMC10634023; doi:10.1186/s40621-023-00462-5)
Supplement: Supplementary file 3 — Additional file 3. Crime category frequency table -- frequencies of legal handgun purchasers in the cohort by specified criminal history and outcome. [file 40621_2023_462_MOESM3_ESM.docx]

Additional Files 3. Frequencies of arrests by crime categories.

| UCR+ Category | Purchasers with at least one prior arrest or no criminal history  *N* | Purchasers with at least one subsequent arrest for a CIV offense and specified criminal history  *N* (%) | Purchasers with at least one subsequent arrest for a firearm-related violent offense and specified criminal history  *N* (%) | Purchasers with at least one subsequent arrest for any violent offense and specified criminal history  *N* (%) |
| --- | --- | --- | --- | --- |
| No criminal history | 66,511 | 1206 (1.8%) | 332 (0.5%) | 2029 (3.1%) |
| Other crimes | 3,035 | 285 (9.4%) | 70 (2.3%) | 468 (15.4%) |
| Weapons | 2,887 | 265 (9.2%) | 76 (2.6%) | 421 (14.6%) |
| Theft | 2,647 | 236 (8.9%) | 69 (2.6%) | 406 (15.3%) |
| Other Simple assaults | 2,518 | 286 (11.4%) | 70 (2.8%) | 453 (18.0%) |
| DUI | 2465 | 213 (8.6%) | 64 (2.6%) | 349 (14.2%) |
| Drug abuse | 2,432 | 217 (8.9%) | 58 (2.4%) | 352 (14.5%) |
| Vehicle violations | 2,430 | 250 (10.3%) | 65 (1.9%) | 427 (17.8%) |
| Aggravated assault | 1,946 | 228 (11.7%) | 60 (3.1%) | 338 (19.9%) |
| Burglary | 1,468 | 130 (8.9%) | 41 (2.8%) | 212 (14.4%) |
| Disorderly conduct | 1403 | 120 (8.6%) | 29 (2.1%) | 188 (13.4%) |
| Vandalism | 1,133 | 104 (9.2%) | 26 (2.3%) | 180 (15.9%) |
| Stolen property | 1,098 | 107 (9.7%) | 29 (2.6%) | 174 (15.8%) |
| Vehicle theft | 667 | 61 (9.1%) | 18 (2.7%) | 115 (17.2%) |
| Fraud | 494 | 47 (9.5%) | 14 (2.8%) | 83 (16.8%) |
| Prostitution | 451 | 31 (6.9%) | 10 (2.2%) | 58 (12.9%) |
| Forgery | 369 | 42 (11.4%) | 14 (3.8%) | 74 (20.1%) |
| Robbery | 308 | 45 (14.6%) | 12 (3.9%) | 64 (20.8%) |
| Sex offenses | 284 | 27 (9.5%) | 6 (2.1%) | 42 (14.8%) |
| Liquor laws | 214 | 19 (8.9%) | 1 (0.5%) | 31 (14.5%) |
| Family and children | 204 | 23 (11.3%) | 8 (3.9%) | 34 (16.7%) |
| Drunkenness | 194 | 23 (11.9%) | 3 (1.5%) | 31 (16.0%) |
| Vagrancy | 174 | 14 (8.0%) | 5 (2.9%) | 26 (14.9%) |
| Embezzlement | 90 | 6 (6.7%) | 3 (3.3%) | 12 (13.3%) |
| Homicide | 81 | 9 (11.1%) | 4 (4.9%) | 17 (21.0%) |
| Rape | 66 | 5 (7.6%) | 3 (4.5%) | 10 (15.1%) |
| Arson | 42 | 5 (11.9%) | 3 (7.1%) | 6 (14.3%) |
| Gambling | 16 | 2 (12.5%) | 2 (12.5%) | 3 (18.8%) |

Table A1.1. Frequencies of legal handgun purchasers in the cohort by specified criminal history and outcome.
