# Supplementary figures and images for: Importance of categories of crime for predicting future violent crime among handgun purchasers in California
Source: Inj Epidemiol. 2023 Nov 9;10:57. doi: 10.1186/s40621-023-00462-5 (PMC10634023; doi:10.1186/s40621-023-00462-5)

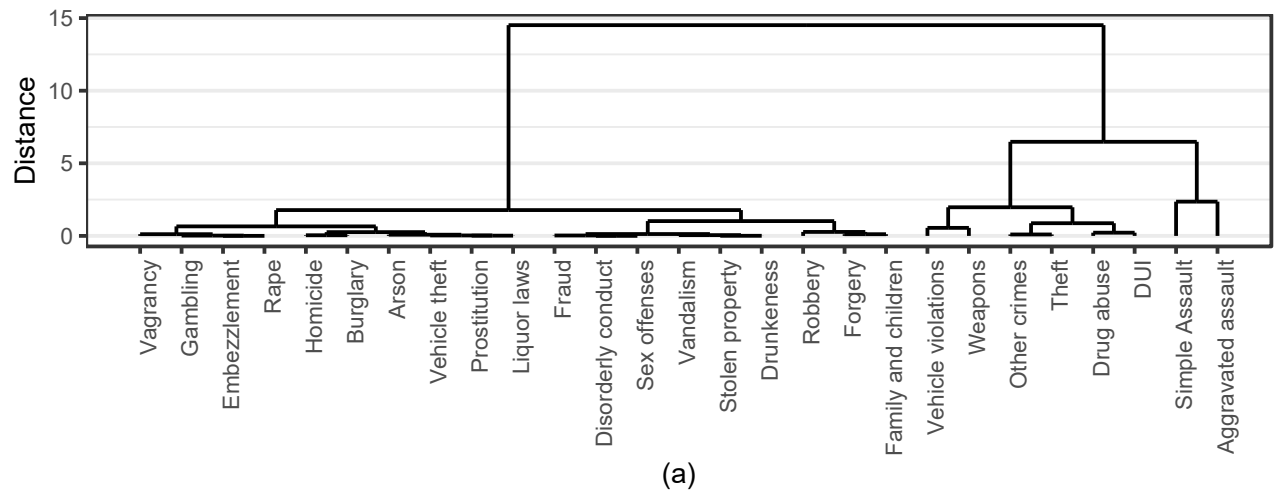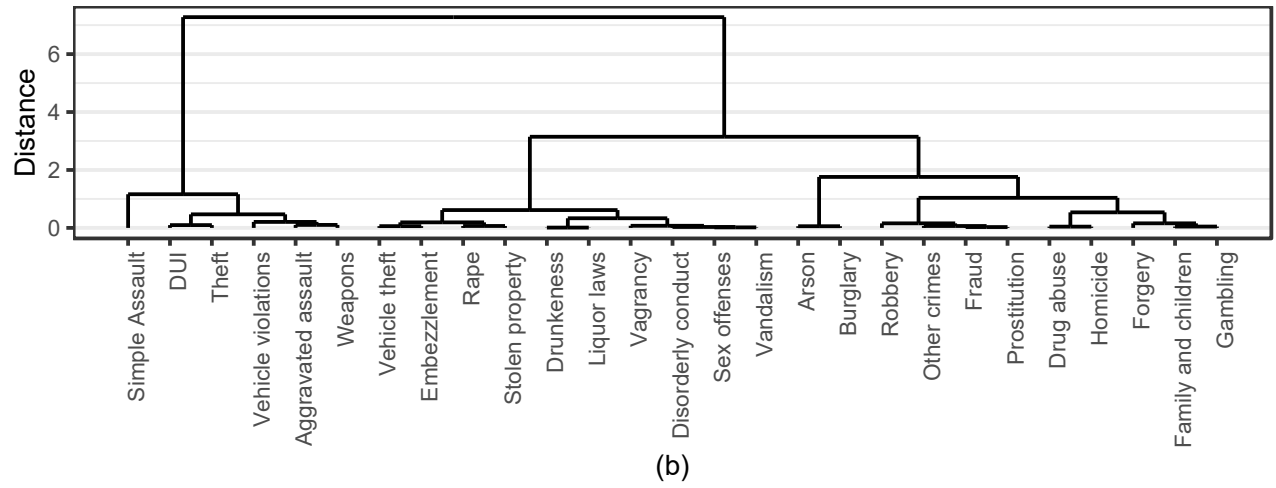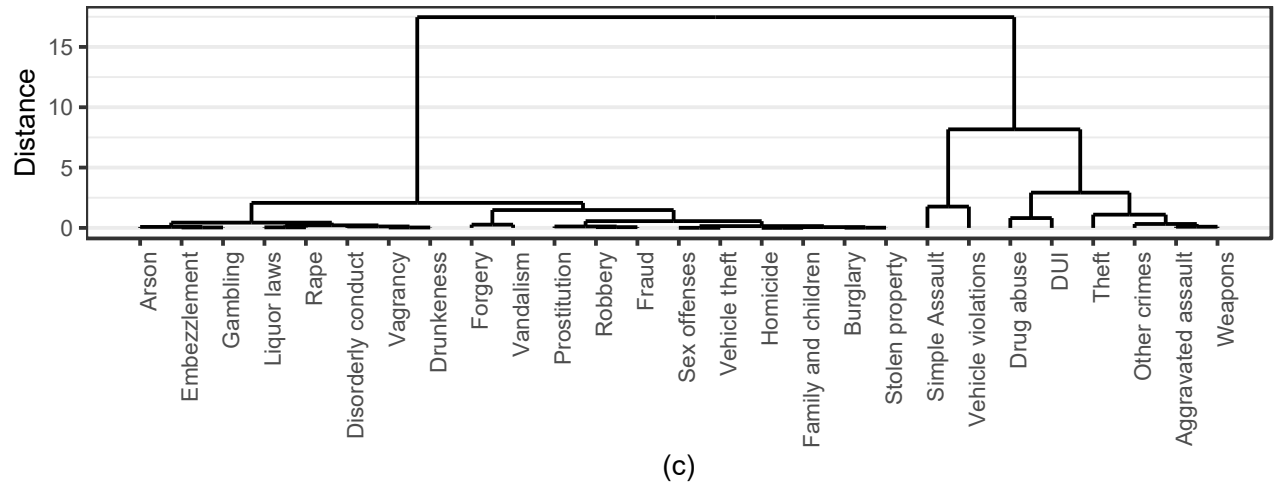

Supplement: Supplementary file 4 — Additional file 4. Hierarchical clustering of crime categories by relative importance–dendrograms of crime categories clustered by relative importance for arrest for a Crime Index-listed violent offense, arrest for a firearm-related violent offense, and arrest for any violent offense. [file 40621_2023_462_MOESM4_ESM.pdf]
