# Supplementary material for: Importance of categories of crime for predicting future violent crime among handgun purchasers in California
Source: Inj Epidemiol. 2023 Nov 9;10:57. doi: 10.1186/s40621-023-00462-5 (PMC10634023; doi:10.1186/s40621-023-00462-5)
Supplement: Supplementary file 5 — Additional file 5. Tables of survival regression model coefficients–tables of regression coefficients for the adjusted Cox proportional hazards models for the primary and secondary outcomes. [file 40621_2023_462_MOESM5_ESM.docx]

Additional File 5. Adjusted hazard ratios for subsequent arrest for a violent crime.

|  | CIV | Firearm violence | Any violence |
| --- | --- | --- | --- |
| Criminal history | Estimate (95% CI) | Estimate (95% CI) | Estimate (95% CI) |
| Simple assault |  |  |  |
| Only simple assault | 4.0 (2.8, 5.9) | 4.6 (2.4, 9.0) | 3.7 (2.7, 5.0) |
| Simple assault and other categories | 7.0 (6.1, 8.0) | 5.5 (4.2, 7.3) | 6.9 (6.2, 7.7) |
| Aggravated assault |  |  |  |
| Only aggravated assault | 3.0 (1.9, 4.7) | 3.2 (1.4, 7.3) | 3.7 (2.7, 5.1) |
| Aggravated assault and other categories | 7.5 (6.4, 8.7) | 6.4 (4.8, 8.5) | 6.7 (6.0, 7.6) |
| Vehicle |  |  |  |
| Only vehicle | 2.6 (1.6, 4.4) | 2.5 (0.9, 6.8) | 3.9 (2.8, 5.5) |
| Vehicle and other categories | 5.8 (5.0, 6.7) | 5.1 (3.9, 6.7) | 6.1 (5.5, 6.8) |
| Weapons |  |  |  |
| Only weapons | 3.4 (2.6, 4.5) | 3.6 (2.1, 6.0) | 3.1 (2.5, 3.9) |
| Weapons and other categories | 5.4 (4.7, 6.3) | 5.2 (3.9, 6.8) | 5.5 (4.9, 6.2) |
| Other crimes (UCR 26) |  |  |  |
| Only UCR 26 | 2.6 (1.7, 4.0) | -- | 2.9 (2.1, 4.0) |
| UCR 26 and other categories | 5.5 (4.8, 6.3) | -- | 5.5 (4.9, 6.2) |
| Theft |  |  |  |
| Only theft | 2.6 (1.8, 3.6) | 2.0 (1.0, 4.1) | 3.0 (2.4, 3.8) |
| Theft and other categories | 5.6 (4.8, 6.5) | 5.8 (4.4, 7.7) | 5.9 (5.2, 6.6) |
| Drug abuse |  |  |  |
| Only drug abuse | 3.2 (2.3, 4.4) | -- | 2.9 (2.3, 3.8) |
| Drug abuse and other categories | 5.7 (4.9. 6.7) | -- | 5.9 (5.2, 6.6) |
| DUI |  |  |  |
| Only DUI | 2.9 (2.1, 4.1) | 3.0 (1.6, 5.6) | 3.4 (2.6, 4.3) |
| DUI and other categories | 5.6 (4.8, 6.6) | 5.8 (4.3, 7.8) | 5.7 (5.0, 6.4) |

Table A5.1. Adjusted hazard ratios for time to arrest with corresponding 95% family-wise confidence intervals by crime categories with high relative influence.
