# Supplementary material for: Importance of categories of crime for predicting future violent crime among handgun purchasers in California
Source: Inj Epidemiol. 2023 Nov 9;10:57. doi: 10.1186/s40621-023-00462-5 (PMC10634023; doi:10.1186/s40621-023-00462-5)
Supplement: Supplementary file 6 — Additional file 6. Estimated adjusted hazard ratios and corresponding family-wise 95% confidence intervals table–estimates and Bonferroni-adjusted confidence interval values corresponding to those shown in Figure 3. [file 40621_2023_462_MOESM6_ESM.docx]

Additional File 6. Tables of model coefficients.

| Variable | coef | exp(coef) | se(coef) | z | p |
| --- | --- | --- | --- | --- | --- |
| Non-simple assault only | 1.264 | 3.538 | 0.047 | 26.62 | 0.000 |
| Simple assault only | 1.404 | 4.071 | 0.192 | 7.330 | 0.000 |
| Simple assault and non-simple assault | 1.943 | 6.980 | 0.070 | 27.90 | 0.000 |
| Male | 0.397 | 1.488 | 0.097 | 4.100 | 0.000 |
| Age at index purchase | -0.046 | 0.955 | 0.003 | -16.15 | 0.000 |
| Num. guns purchased prior to index | -0.010 | 0.990 | 0.005 | -1.910 | 0.056 |
| Census tract population | -0.000 | 1.000 | 0.000 | -0.550 | 0.580 |
| Census tract prop. male | 0.003 | 1.003 | 0.008 | 0.380 | 0.700 |
| Census tract SES index | -0.242 | 0.785 | 0.032 | -7.560 | 0.000 |
| Census tract pop. per sq.mi. | 0.000 | 1.000 | 0.000 | 0.820 | 0.410 |
| Census tract prop. age 20-24 of 20-44 | 0.004 | 1.004 | 0.005 | 0.820 | 0.410 |
| Census tract bars/pubs per sq.mi. | -0.009 | 0.991 | 0.007 | -1.170 | 0.240 |
| Census tract off-premise per sq.mi. | 0.004 | 1.004 | 0.004 | 1.010 | 0.310 |
| Census tract restrnt br/wn per sq.mi. | 0.003 | 1.003 | 0.006 | 0.420 | 0.670 |
| Census tract restrnt sprt per sq.mi. | -0.003 | 0.997 | 0.004 | -0.610 | 0.540 |
| County population | -0.000 | 1.000 | 0.000 | -1.830 | 0.067 |
| County viol. crime per 100k | 0.000 | 1.000 | 0.000 | 1.430 | 0.150 |
| County property crime per 100k | -0.000 | 1.000 | 0.000 | -1.000 | 0.320 |
| County firearm suicide / suicide | -0.457 | 0.633 | 0.503 | -0.910 | 0.360 |

Table A6.1. Cox proportional hazards model coefficients for the model with subsequent arrest for a Crime Index-listed violent crime as the outcome and prior arrest for simple assault as the primary exposure.

| Variable | coef | exp(coef) | se(coef) | z | p |
| --- | --- | --- | --- | --- | --- |
| Non-agg. assault only | 1.296 | 3.655 | 0.046 | 27.96 | 0.000 |
| Agg. assault only | 1.090 | 2.975 | 0.232 | 4.700 | 0.000 |
| Agg. assault and non-agg. assault | 2.012 | 7.481 | 0.076 | 26.43 | 0.000 |
| Male | 0.395 | 1.485 | 0.097 | 4.080 | 0.000 |
| Age at index purchase | -0.046 | 0.955 | 0.003 | -16.11 | 0.000 |
| Num. guns purchased prior to index | -0.010 | 0.990 | 0.005 | -2.020 | 0.043 |
| Census tract population | -0.000 | 1.000 | 0.000 | -0.610 | 0.540 |
| Census tract prop. male | 0.003 | 1.003 | 0.008 | 0.360 | 0.720 |
| Census tract SES index | -0.237 | 0.789 | 0.032 | -7.440 | 0.000 |
| Census tract pop. per sq.mi. | 0.000 | 1.000 | 0.000 | 0.850 | 0.400 |
| Census tract prop. age 20-24 of 20-44 | 0.004 | 1.004 | 0.005 | 0.780 | 0.440 |
| Census tract bars/pubs per sq.mi. | -0.009 | 0.991 | 0.007 | -1.200 | 0.230 |
| Census tract off-premise per sq.mi. | 0.004 | 1.004 | 0.004 | 0.960 | 0.340 |
| Census tract restrnt br/wn per sq.mi. | 0.003 | 1.003 | 0.006 | 0.440 | 0.660 |
| Census tract restrnt sprt per sq.mi. | -0.002 | 0.998 | 0.004 | -0.570 | 0.570 |
| County population | -0.000 | 1.000 | 0.000 | -1.960 | 0.050 |
| County viol. crime per 100k | 0.001 | 1.001 | 0.000 | 1.470 | 0.140 |
| County property crime per 100k | -0.000 | 1.000 | 0.000 | -1.090 | 0.280 |
| County firearm suicide / suicide | -0.439 | 0.645 | 0.501 | -0.880 | 0.380 |

Table A6.2. Cox proportional hazards model coefficients for the model with subsequent arrest for a Crime Index-listed violent crime as the outcome and prior arrest for aggravated assault as the primary exposure.

| Variable | coef | exp(coef) | se(coef) | z | p |
| --- | --- | --- | --- | --- | --- |
| Non-vehi. violation only | 1.323 | 3.756 | 0.047 | 28.31 | 0.000 |
| Vehi. violation only | 0.967 | 2.629 | 0.260 | 3.710 | 0.000 |
| Vehi. violation and non-vehi. violation | 1.758 | 5.800 | 0.072 | 24.35 | 0.000 |
| Male | 0.401 | 1.493 | 0.097 | 4.130 | 0.000 |
| Age at index purchase | -0.045 | 0.956 | 0.003 | -15.70 | 0.000 |
| Num. guns purchased prior to index | -0.010 | 0.990 | 0.005 | -1.940 | 0.053 |
| Census tract population | -0.000 | 1.000 | 0.000 | -0.540 | 0.590 |
| Census tract prop. male | 0.003 | 1.003 | 0.008 | 0.400 | 0.690 |
| Census tract SES index | -0.243 | 0.785 | 0.032 | -7.590 | 0.000 |
| Census tract pop. per sq.mi. | 0.000 | 1.000 | 0.000 | 0.790 | 0.430 |
| Census tract prop. age 20-24 of 20-44 | 0.003 | 1.003 | 0.005 | 0.720 | 0.470 |
| Census tract bars/pubs per sq.mi. | -0.009 | 0.991 | 0.008 | -1.220 | 0.220 |
| Census tract off-premise per sq.mi. | 0.004 | 1.004 | 0.004 | 1.030 | 0.300 |
| Census tract restrnt br/wn per sq.mi. | 0.003 | 1.003 | 0.006 | 0.430 | 0.670 |
| Census tract restrnt sprt per sq.mi. | -0.002 | 0.998 | 0.004 | -0.590 | 0.560 |
| County population | -0.000 | 1.000 | 0.000 | -2.000 | 0.046 |
| County viol. crime per 100k | 0.001 | 1.001 | 0.000 | 1.480 | 0.140 |
| County property crime per 100k | -0.000 | 1.000 | 0.000 | -1.020 | 0.310 |
| County firearm suicide / suicide | -0.406 | 0.666 | 0.502 | -0.810 | 0.420 |

Table A6.3. Cox proportional hazards model coefficients for the model with subsequent arrest for a Crime Index-listed violent crime as the outcome and prior arrest for vehicle violation as the primary exposure.

| Variable | coef | exp(coef) | se(coef) | z | p |
| --- | --- | --- | --- | --- | --- |
| Non-weapons only | 1.343 | 3.832 | 0.047 | 28.60 | 0.000 |
| Weapons only | 1.223 | 3.398 | 0.146 | 8.360 | 0.000 |
| Weapons and non-weapons | 1.694 | 5.440 | 0.075 | 22.51 | 0.000 |
| Male | 0.397 | 1.488 | 0.097 | 4.100 | 0.000 |
| Age at index purchase | -0.045 | 0.956 | 0.003 | -15.69 | 0.000 |
| Num. guns purchased prior to index | -0.011 | 0.989 | 0.005 | -2.120 | 0.034 |
| Census tract population | -0.000 | 1.000 | 0.000 | -0.610 | 0.540 |
| Census tract prop. male | 0.003 | 1.003 | 0.008 | 0.400 | 0.690 |
| Census tract SES index | -0.239 | 0.787 | 0.032 | -7.490 | 0.000 |
| Census tract pop. per sq.mi. | 0.000 | 1.000 | 0.000 | 0.880 | 0.380 |
| Census tract prop. age 20-24 of 20-44 | 0.004 | 1.004 | 0.005 | 0.800 | 0.420 |
| Census tract bars/pubs per sq.mi. | -0.009 | 0.991 | 0.008 | -1.240 | 0.220 |
| Census tract off-premise per sq.mi. | 0.004 | 1.004 | 0.004 | 1.000 | 0.320 |
| Census tract restrnt br/wn per sq.mi. | 0.002 | 1.002 | 0.006 | 0.350 | 0.720 |
| Census tract restrnt sprt per sq.mi. | -0.002 | 0.998 | 0.004 | -0.500 | 0.620 |
| County population | -0.000 | 1.000 | 0.000 | -2.080 | 0.038 |
| County viol. crime per 100k | 0.001 | 1.001 | 0.000 | 1.580 | 0.110 |
| County property crime per 100k | -0.000 | 1.000 | 0.000 | -1.140 | 0.250 |
| County firearm suicide / suicide | -0.393 | 0.675 | 0.502 | -0.780 | 0.430 |

Table A6.4. Cox proportional hazards model coefficients for the model with subsequent arrest for a Crime Index-listed violent crime as the outcome and prior arrest for weapon crime as the primary exposure.

| Variable | coef | exp(coef) | se(coef) | z | p |
| --- | --- | --- | --- | --- | --- |
| Non-other crimes only | 1.329 | 3.779 | 0.047 | 28.04 | 0.000 |
| Other crimes only | 0.963 | 2.620 | 0.211 | 4.570 | 0.000 |
| Other crimes and non-other crimes | 1.698 | 5.461 | 0.069 | 24.54 | 0.000 |
| Male | 0.408 | 1.503 | 0.097 | 4.200 | 0.000 |
| Age at index purchase | -0.045 | 0.956 | 0.003 | -15.76 | 0.000 |
| Num. guns purchased prior to index | -0.010 | 0.990 | 0.005 | -1.960 | 0.050 |
| Census tract population | -0.000 | 1.000 | 0.000 | -0.520 | 0.600 |
| Census tract prop. male | 0.003 | 1.003 | 0.008 | 0.380 | 0.700 |
| Census tract SES index | -0.243 | 0.784 | 0.032 | -7.600 | 0.000 |
| Census tract pop. per sq.mi. | 0.000 | 1.000 | 0.000 | 0.750 | 0.450 |
| Census tract prop. age 20-24 of 20-44 | 0.004 | 1.004 | 0.005 | 0.770 | 0.440 |
| Census tract bars/pubs per sq.mi. | -0.009 | 0.991 | 0.008 | -1.230 | 0.220 |
| Census tract off-premise per sq.mi. | 0.004 | 1.004 | 0.004 | 1.060 | 0.290 |
| Census tract restrnt br/wn per sq.mi. | 0.002 | 1.002 | 0.006 | 0.350 | 0.720 |
| Census tract restrnt sprt per sq.mi. | -0.002 | 0.998 | 0.004 | -0.520 | 0.600 |
| County population | -0.000 | 1.000 | 0.000 | -2.050 | 0.040 |
| County viol. crime per 100k | 0.001 | 1.001 | 0.000 | 1.610 | 0.110 |
| County property crime per 100k | -0.000 | 1.000 | 0.000 | -1.120 | 0.260 |
| County firearm suicide / suicide | -0.420 | 0.657 | 0.502 | -0.840 | 0.400 |

Table A6.5. Cox proportional hazards model coefficients for the model with subsequent arrest for a Crime Index-listed violent crime as the outcome and prior arrest for other crime as the primary exposure.

| Variable | coef | exp(coef) | se(coef) | z | p |
| --- | --- | --- | --- | --- | --- |
| Non-theft only | 1.359 | 3.892 | 0.047 | 29.15 | 0.000 |
| Theft only | 0.942 | 2.566 | 0.167 | 5.630 | 0.000 |
| Theft and non-theft | 1.723 | 5.602 | 0.077 | 22.30 | 0.000 |
| Male | 0.407 | 1.502 | 0.097 | 4.190 | 0.000 |
| Age at index purchase | -0.045 | 0.956 | 0.003 | -15.71 | 0.000 |
| Num. guns purchased prior to index | -0.010 | 0.990 | 0.005 | -1.960 | 0.050 |
| Census tract population | -0.000 | 1.000 | 0.000 | -0.590 | 0.550 |
| Census tract prop. male | 0.003 | 1.003 | 0.008 | 0.420 | 0.680 |
| Census tract SES index | -0.243 | 0.784 | 0.032 | -7.620 | 0.000 |
| Census tract pop. per sq.mi. | 0.000 | 1.000 | 0.000 | 0.890 | 0.370 |
| Census tract prop. age 20-24 of 20-44 | 0.003 | 1.003 | 0.005 | 0.710 | 0.480 |
| Census tract bars/pubs per sq.mi. | -0.010 | 0.990 | 0.008 | -1.270 | 0.200 |
| Census tract off-premise per sq.mi. | 0.004 | 1.004 | 0.004 | 0.960 | 0.330 |
| Census tract restrnt br/wn per sq.mi. | 0.002 | 1.002 | 0.006 | 0.410 | 0.680 |
| Census tract restrnt sprt per sq.mi. | -0.002 | 0.998 | 0.004 | -0.540 | 0.590 |
| County population | -0.000 | 1.000 | 0.000 | -2.090 | 0.037 |
| County viol. crime per 100k | 0.001 | 1.001 | 0.000 | 1.580 | 0.110 |
| County property crime per 100k | -0.000 | 1.000 | 0.000 | -1.130 | 0.260 |
| County firearm suicide / suicide | -0.402 | 0.669 | 0.502 | -0.800 | 0.420 |

Table A6.6. Cox proportional hazards model coefficients for the model with subsequent arrest for a Crime Index-listed violent crime as the outcome and prior arrest for theft as the primary exposure.

| Variable | coef | exp(coef) | se(coef) | z | p |
| --- | --- | --- | --- | --- | --- |
| Non-drug only | 1.350 | 3.857 | 0.046 | 29.21 | 0.000 |
| Drug only | 1.151 | 3.160 | 0.170 | 6.790 | 0.000 |
| Drug and non-drug | 1.738 | 5.683 | 0.081 | 21.50 | 0.000 |
| Male | 0.407 | 1.502 | 0.097 | 4.190 | 0.000 |
| Age at index purchase | -0.045 | 0.956 | 0.003 | -15.83 | 0.000 |
| Num. guns purchased prior to index | -0.010 | 0.990 | 0.005 | -1.900 | 0.057 |
| Census tract population | -0.000 | 1.000 | 0.000 | -0.580 | 0.560 |
| Census tract prop. male | 0.003 | 1.003 | 0.008 | 0.350 | 0.720 |
| Census tract SES index | -0.243 | 0.785 | 0.032 | -7.600 | 0.000 |
| Census tract pop. per sq.mi. | 0.000 | 1.000 | 0.000 | 0.850 | 0.390 |
| Census tract prop. age 20-24 of 20-44 | 0.004 | 1.004 | 0.005 | 0.750 | 0.450 |
| Census tract bars/pubs per sq.mi. | -0.009 | 0.991 | 0.008 | -1.240 | 0.220 |
| Census tract off-premise per sq.mi. | 0.004 | 1.004 | 0.004 | 1.060 | 0.290 |
| Census tract restrnt br/wn per sq.mi. | 0.002 | 1.002 | 0.006 | 0.390 | 0.700 |
| Census tract restrnt sprt per sq.mi. | -0.002 | 0.998 | 0.004 | -0.550 | 0.580 |
| County population | -0.000 | 1.000 | 0.000 | -2.030 | 0.042 |
| County viol. crime per 100k | 0.001 | 1.001 | 0.000 | 1.520 | 0.130 |
| County property crime per 100k | -0.000 | 1.000 | 0.000 | -1.070 | 0.290 |
| County firearm suicide / suicide | -0.424 | 0.655 | 0.502 | -0.840 | 0.400 |

Table A6.7. Cox proportional hazards model coefficients for the model with subsequent arrest for a Crime Index-listed violent crime as the outcome and prior arrest for drug abuse crime as the primary exposure.

| Variable | coef | exp(coef) | se(coef) | z | p |
| --- | --- | --- | --- | --- | --- |
| Non-DUI only | 1.357 | 3.884 | 0.046 | 29.39 | 0.000 |
| DUI only | 1.074 | 2.926 | 0.172 | 6.250 | 0.000 |
| DUI and non-DUI | 1.726 | 5.616 | 0.081 | 21.24 | 0.000 |
| Male | 0.403 | 1.497 | 0.097 | 4.160 | 0.000 |
| Age at index purchase | -0.045 | 0.956 | 0.003 | -15.85 | 0.000 |
| Num. guns purchased prior to index | -0.010 | 0.990 | 0.005 | -1.910 | 0.057 |
| Census tract population | -0.000 | 1.000 | 0.000 | -0.560 | 0.580 |
| Census tract prop. male | 0.003 | 1.003 | 0.008 | 0.360 | 0.720 |
| Census tract SES index | -0.243 | 0.784 | 0.032 | -7.620 | 0.000 |
| Census tract pop. per sq.mi. | 0.000 | 1.000 | 0.000 | 0.840 | 0.400 |
| Census tract prop. age 20-24 of 20-44 | 0.003 | 1.003 | 0.005 | 0.720 | 0.470 |
| Census tract bars/pubs per sq.mi. | -0.009 | 0.991 | 0.008 | -1.230 | 0.220 |
| Census tract off-premise per sq.mi. | 0.004 | 1.004 | 0.004 | 1.080 | 0.280 |
| Census tract restrnt br/wn per sq.mi. | 0.002 | 1.002 | 0.006 | 0.410 | 0.680 |
| Census tract restrnt sprt per sq.mi. | -0.002 | 0.998 | 0.004 | -0.580 | 0.560 |
| County population | -0.000 | 1.000 | 0.000 | -2.060 | 0.039 |
| County viol. crime per 100k | 0.001 | 1.001 | 0.000 | 1.550 | 0.120 |
| County property crime per 100k | -0.000 | 1.000 | 0.000 | -1.120 | 0.260 |
| County firearm suicide / suicide | -0.440 | 0.644 | 0.501 | -0.880 | 0.380 |

Table A6.8. Cox proportional hazards model coefficients for the model with subsequent arrest for a Crime Index-listed violent crime as the outcome and prior arrest for driving under the influence as the primary exposure.

| Variable | coef | exp(coef) | se(coef) | z | p |
| --- | --- | --- | --- | --- | --- |
| Non-simple assault only | 1.207 | 3.342 | 0.091 | 13.23 | 0.000 |
| Simple assault only | 1.536 | 4.645 | 0.339 | 4.540 | 0.000 |
| Simple assault and non-simple assault | 1.706 | 5.505 | 0.142 | 12.02 | 0.000 |
| Male | 0.570 | 1.768 | 0.202 | 2.820 | 0.005 |
| Age at index purchase | -0.035 | 0.965 | 0.005 | -6.560 | 0.000 |
| Num. guns purchased prior to index | -0.001 | 0.999 | 0.007 | -0.110 | 0.910 |
| Census tract population | 0.000 | 1.000 | 0.000 | 0.120 | 0.900 |
| Census tract prop. male | -0.001 | 0.999 | 0.015 | -0.040 | 0.970 |
| Census tract SES index | -0.286 | 0.751 | 0.063 | -4.500 | 0.000 |
| Census tract pop. per sq.mi. | 0.000 | 1.000 | 0.000 | 0.400 | 0.690 |
| Census tract prop. age 20-24 of 20-44 | -0.007 | 0.993 | 0.010 | -0.680 | 0.500 |
| Census tract bars/pubs per sq.mi. | -0.009 | 0.991 | 0.016 | -0.580 | 0.560 |
| Census tract off-premise per sq.mi. | 0.004 | 1.004 | 0.008 | 0.480 | 0.630 |
| Census tract restrnt br/wn per sq.mi. | -0.012 | 0.988 | 0.013 | -0.930 | 0.350 |
| Census tract restrnt sprt per sq.mi. | 0.005 | 1.005 | 0.008 | 0.600 | 0.550 |
| County population | -0.000 | 1.000 | 0.000 | -0.590 | 0.550 |
| County viol. crime per 100k | 0.001 | 1.001 | 0.001 | 0.900 | 0.370 |
| County property crime per 100k | -0.000 | 1.000 | 0.000 | -0.720 | 0.470 |
| County firearm suicide / suicide | -1.190 | 0.304 | 0.877 | -1.360 | 0.170 |

Table A6.9. Cox proportional hazards model coefficients for the model with subsequent arrest for a firearm-related violent crime as the outcome and prior arrest for simple assault as the primary exposure.

| Variable | coef | exp(coef) | se(coef) | z | p |
| --- | --- | --- | --- | --- | --- |
| Non-agg. assault only | 1.212 | 3.360 | 0.090 | 13.49 | 0.000 |
| Agg. assault only | 1.176 | 3.241 | 0.413 | 2.850 | 0.004 |
| Agg. assault and non-agg. assault | 1.853 | 6.379 | 0.149 | 12.44 | 0.000 |
| Male | 0.566 | 1.762 | 0.202 | 2.800 | 0.005 |
| Age at index purchase | -0.036 | 0.965 | 0.005 | -6.600 | 0.000 |
| Num. guns purchased prior to index | -0.001 | 0.999 | 0.007 | -0.160 | 0.880 |
| Census tract population | 0.000 | 1.000 | 0.000 | 0.100 | 0.920 |
| Census tract prop. male | -0.001 | 0.999 | 0.015 | -0.060 | 0.960 |
| Census tract SES index | -0.281 | 0.755 | 0.063 | -4.430 | 0.000 |
| Census tract pop. per sq.mi. | 0.000 | 1.000 | 0.000 | 0.400 | 0.690 |
| Census tract prop. age 20-24 of 20-44 | -0.007 | 0.993 | 0.010 | -0.690 | 0.490 |
| Census tract bars/pubs per sq.mi. | -0.009 | 0.991 | 0.016 | -0.580 | 0.560 |
| Census tract off-premise per sq.mi. | 0.004 | 1.004 | 0.008 | 0.450 | 0.650 |
| Census tract restrnt br/wn per sq.mi. | -0.012 | 0.988 | 0.013 | -0.910 | 0.360 |
| Census tract restrnt sprt per sq.mi. | 0.005 | 1.005 | 0.008 | 0.610 | 0.540 |
| County population | -0.000 | 1.000 | 0.000 | -0.660 | 0.510 |
| County viol. crime per 100k | 0.001 | 1.001 | 0.001 | 0.910 | 0.360 |
| County property crime per 100k | -0.000 | 1.000 | 0.000 | -0.760 | 0.450 |
| County firearm suicide / suicide | -1.190 | 0.304 | 0.876 | -1.360 | 0.170 |

Table A6.10. Cox proportional hazards model coefficients for the model with subsequent arrest for a firearm-related violent crime as the outcome and prior arrest for aggravated assault as the primary exposure.

| Variable | coef | exp(coef) | se(coef) | z | p |
| --- | --- | --- | --- | --- | --- |
| Non-vehi. violation only | 1.239 | 3.453 | 0.091 | 13.69 | 0.000 |
| Vehi. violation only | 0.932 | 2.539 | 0.504 | 1.850 | 0.064 |
| Vehi. violation and non-vehi. violation | 1.633 | 5.120 | 0.141 | 11.59 | 0.000 |
| Male | 0.571 | 1.770 | 0.202 | 2.830 | 0.005 |
| Age at index purchase | -0.034 | 0.966 | 0.005 | -6.410 | 0.000 |
| Num. guns purchased prior to index | -0.001 | 0.999 | 0.007 | -0.110 | 0.910 |
| Census tract population | 0.000 | 1.000 | 0.000 | 0.130 | 0.900 |
| Census tract prop. male | -0.000 | 1.000 | 0.015 | -0.030 | 0.970 |
| Census tract SES index | -0.286 | 0.751 | 0.063 | -4.510 | 0.000 |
| Census tract pop. per sq.mi. | 0.000 | 1.000 | 0.000 | 0.370 | 0.710 |
| Census tract prop. age 20-24 of 20-44 | -0.007 | 0.993 | 0.010 | -0.710 | 0.480 |
| Census tract bars/pubs per sq.mi. | -0.010 | 0.990 | 0.016 | -0.620 | 0.540 |
| Census tract off-premise per sq.mi. | 0.004 | 1.004 | 0.008 | 0.490 | 0.630 |
| Census tract restrnt br/wn per sq.mi. | -0.012 | 0.988 | 0.013 | -0.900 | 0.370 |
| Census tract restrnt sprt per sq.mi. | 0.005 | 1.005 | 0.008 | 0.600 | 0.550 |
| County population | -0.000 | 1.000 | 0.000 | -0.680 | 0.500 |
| County viol. crime per 100k | 0.001 | 1.001 | 0.001 | 0.920 | 0.360 |
| County property crime per 100k | -0.000 | 1.000 | 0.000 | -0.730 | 0.470 |
| County firearm suicide / suicide | -1.159 | 0.314 | 0.876 | -1.320 | 0.190 |

Table A6.11. Cox proportional hazards model coefficients for the model with subsequent arrest for a firearm-related violent crime as the outcome and prior arrest for vehicle violations as the primary exposure.

| Variable | coef | exp(coef) | se(coef) | z | p |
| --- | --- | --- | --- | --- | --- |
| Non-weapons only | 1.233 | 3.430 | 0.092 | 13.42 | 0.000 |
| Weapons only | 1.268 | 3.553 | 0.265 | 4.780 | 0.000 |
| Weapons and non-weapons | 1.640 | 5.154 | 0.142 | 11.53 | 0.000 |
| Male | 0.562 | 1.754 | 0.202 | 2.780 | 0.005 |
| Age at index purchase | -0.035 | 0.966 | 0.005 | -6.430 | 0.000 |
| Num. guns purchased prior to index | -0.002 | 0.998 | 0.008 | -0.250 | 0.800 |
| Census tract population | 0.000 | 1.000 | 0.000 | 0.200 | 0.840 |
| Census tract prop. male | -0.001 | 0.999 | 0.015 | -0.060 | 0.950 |
| Census tract SES index | -0.282 | 0.755 | 0.063 | -4.440 | 0.000 |
| Census tract pop. per sq.mi. | 0.000 | 1.000 | 0.000 | 0.430 | 0.670 |
| Census tract prop. age 20-24 of 20-44 | -0.007 | 0.993 | 0.010 | -0.720 | 0.470 |
| Census tract bars/pubs per sq.mi. | -0.010 | 0.990 | 0.016 | -0.640 | 0.530 |
| Census tract off-premise per sq.mi. | 0.004 | 1.004 | 0.008 | 0.480 | 0.630 |
| Census tract restrnt br/wn per sq.mi. | -0.012 | 0.988 | 0.013 | -0.930 | 0.350 |
| Census tract restrnt sprt per sq.mi. | 0.005 | 1.005 | 0.008 | 0.650 | 0.520 |
| County population | -0.000 | 1.000 | 0.000 | -0.890 | 0.370 |
| County viol. crime per 100k | 0.001 | 1.001 | 0.001 | 1.120 | 0.260 |
| County property crime per 100k | -0.000 | 1.000 | 0.000 | -0.910 | 0.360 |
| County firearm suicide / suicide | -1.082 | 0.339 | 0.849 | -1.270 | 0.200 |

Table A6.12. Cox proportional hazards model coefficients for the model with subsequent arrest for a firearm-related violent crime as the outcome and prior arrest for weapon crime as the primary exposure.

| Variable | coef | exp(coef) | se(coef) | z | p |
| --- | --- | --- | --- | --- | --- |
| Non-theft only | 1.239 | 3.451 | 0.091 | 13.60 | 0.000 |
| Theft only | 0.698 | 2.010 | 0.358 | 1.950 | 0.051 |
| Theft and non-theft | 1.765 | 5.844 | 0.141 | 12.49 | 0.000 |
| Male | 0.579 | 1.783 | 0.202 | 2.860 | 0.004 |
| Age at index purchase | -0.035 | 0.966 | 0.005 | -6.440 | 0.000 |
| Num. guns purchased prior to index | -0.001 | 0.999 | 0.007 | -0.120 | 0.910 |
| Census tract population | 0.000 | 1.000 | 0.000 | 0.210 | 0.830 |
| Census tract prop. male | -0.001 | 0.999 | 0.015 | -0.050 | 0.960 |
| Census tract SES index | -0.286 | 0.751 | 0.063 | -4.520 | 0.000 |
| Census tract pop. per sq.mi. | 0.000 | 1.000 | 0.000 | 0.430 | 0.670 |
| Census tract prop. age 20-24 of 20-44 | -0.008 | 0.992 | 0.010 | -0.760 | 0.450 |
| Census tract bars/pubs per sq.mi. | -0.011 | 0.989 | 0.016 | -0.680 | 0.500 |
| Census tract off-premise per sq.mi. | 0.004 | 1.004 | 0.008 | 0.460 | 0.640 |
| Census tract restrnt br/wn per sq.mi. | -0.012 | 0.988 | 0.013 | -0.930 | 0.350 |
| Census tract restrnt sprt per sq.mi. | 0.005 | 1.005 | 0.008 | 0.640 | 0.520 |
| County population | -0.000 | 1.000 | 0.000 | -0.900 | 0.370 |
| County viol. crime per 100k | 0.001 | 1.001 | 0.001 | 1.120 | 0.260 |
| County property crime per 100k | -0.000 | 1.000 | 0.000 | -0.900 | 0.370 |
| County firearm suicide / suicide | -1.098 | 0.333 | 0.848 | -1.290 | 0.200 |

Table A6.13. Cox proportional hazards model coefficients for the model with subsequent arrest for a firearm-related violent crime as the outcome and prior arrest for theft as the primary exposure.

| Variable | coef | exp(coef) | se(coef) | z | p |
| --- | --- | --- | --- | --- | --- |
| Non-DUI only | 1.232 | 3.429 | 0.090 | 13.64 | 0.000 |
| DUI only | 1.100 | 3.003 | 0.322 | 3.420 | 0.001 |
| DUI and non-DUI | 1.761 | 5.820 | 0.149 | 11.80 | 0.000 |
| Male | 0.568 | 1.764 | 0.202 | 2.810 | 0.005 |
| Age at index purchase | -0.035 | 0.965 | 0.005 | -6.580 | 0.000 |
| Num. guns purchased prior to index | -0.001 | 0.999 | 0.007 | -0.070 | 0.940 |
| Census tract population | 0.000 | 1.000 | 0.000 | 0.250 | 0.810 |
| Census tract prop. male | -0.001 | 0.999 | 0.015 | -0.090 | 0.930 |
| Census tract SES index | -0.287 | 0.750 | 0.063 | -4.530 | 0.000 |
| Census tract pop. per sq.mi. | 0.000 | 1.000 | 0.000 | 0.420 | 0.680 |
| Census tract prop. age 20-24 of 20-44 | -0.008 | 0.992 | 0.010 | -0.760 | 0.450 |
| Census tract bars/pubs per sq.mi. | -0.010 | 0.990 | 0.016 | -0.640 | 0.520 |
| Census tract off-premise per sq.mi. | 0.004 | 1.004 | 0.008 | 0.520 | 0.600 |
| Census tract restrnt br/wn per sq.mi. | -0.012 | 0.988 | 0.013 | -0.910 | 0.360 |
| Census tract restrnt sprt per sq.mi. | 0.005 | 1.005 | 0.008 | 0.600 | 0.550 |
| County population | -0.000 | 1.000 | 0.000 | -0.860 | 0.390 |
| County viol. crime per 100k | 0.001 | 1.001 | 0.001 | 1.090 | 0.280 |
| County property crime per 100k | -0.000 | 1.000 | 0.000 | -0.880 | 0.380 |
| County firearm suicide / suicide | -1.159 | 0.314 | 0.849 | -1.360 | 0.170 |

Table A6.14. Cox proportional hazards model coefficients for the model with subsequent arrest for a firearm-related violent crime as the outcome and prior arrest for driving under the influence as the primary exposure.

| Variable | coef | exp(coef) | se(coef) | z | p |
| --- | --- | --- | --- | --- | --- |
| Non-simple assault only | 1.305 | 3.686 | 0.036 | 35.87 | 0.000 |
| Simple assault only | 1.304 | 3.684 | 0.156 | 8.340 | 0.000 |
| Simple assault and non-simple assault | 1.936 | 6.930 | 0.055 | 35.29 | 0.000 |
| Male | 0.378 | 1.459 | 0.074 | 5.100 | 0.000 |
| Age at index purchase | -0.044 | 0.957 | 0.002 | -20.31 | 0.000 |
| Num. guns purchased prior to index | -0.008 | 0.992 | 0.004 | -2.180 | 0.030 |
| Census tract population | -0.000 | 1.000 | 0.000 | -0.830 | 0.410 |
| Census tract prop. male | 0.004 | 1.004 | 0.006 | 0.650 | 0.520 |
| Census tract SES index | -0.213 | 0.808 | 0.025 | -8.520 | 0.000 |
| Census tract pop. per sq.mi. | 0.000 | 1.000 | 0.000 | 1.370 | 0.170 |
| Census tract prop. age 20-24 of 20-44 | -0.002 | 0.998 | 0.004 | -0.590 | 0.560 |
| Census tract bars/pubs per sq.mi. | -0.003 | 0.997 | 0.005 | -0.470 | 0.640 |
| Census tract off-premise per sq.mi. | 0.003 | 1.003 | 0.003 | 1.110 | 0.270 |
| Census tract restrnt br/wn per sq.mi. | -0.005 | 0.995 | 0.004 | -1.030 | 0.300 |
| Census tract restrnt sprt per sq.mi. | 0.001 | 1.001 | 0.003 | 0.500 | 0.620 |
| County population | -0.000 | 1.000 | 0.000 | -1.900 | 0.058 |
| County viol. crime per 100k | 0.000 | 1.000 | 0.000 | 1.010 | 0.310 |
| County property crime per 100k | -0.000 | 1.000 | 0.000 | -1.050 | 0.290 |
| County firearm suicide / suicide | -0.803 | 0.448 | 0.379 | -2.120 | 0.034 |

Table A6.15. Cox proportional hazards model coefficients for the model with subsequent arrest for a any violent crime as the outcome and prior arrest for simple assault as the primary exposure.

| Variable | coef | exp(coef) | se(coef) | z | p |
| --- | --- | --- | --- | --- | --- |
| Non-agg. assault only | 1.347 | 3.845 | 0.035 | 38.04 | 0.000 |
| Agg. assault only | 1.306 | 3.693 | 0.165 | 7.940 | 0.000 |
| Agg. assault and non-agg. assault | 1.907 | 6.734 | 0.063 | 30.49 | 0.000 |
| Male | 0.379 | 1.461 | 0.074 | 5.120 | 0.000 |
| Age at index purchase | -0.044 | 0.957 | 0.002 | -20.13 | 0.000 |
| Num. guns purchased prior to index | -0.009 | 0.992 | 0.004 | -2.290 | 0.022 |
| Census tract population | -0.000 | 1.000 | 0.000 | -0.880 | 0.380 |
| Census tract prop. male | 0.004 | 1.004 | 0.006 | 0.590 | 0.550 |
| Census tract SES index | -0.210 | 0.811 | 0.025 | -8.390 | 0.000 |
| Census tract pop. per sq.mi. | 0.000 | 1.000 | 0.000 | 1.400 | 0.160 |
| Census tract prop. age 20-24 of 20-44 | -0.002 | 0.998 | 0.004 | -0.620 | 0.530 |
| Census tract bars/pubs per sq.mi. | -0.003 | 0.997 | 0.005 | -0.550 | 0.580 |
| Census tract off-premise per sq.mi. | 0.003 | 1.003 | 0.003 | 1.050 | 0.290 |
| Census tract restrnt br/wn per sq.mi. | -0.005 | 0.995 | 0.004 | -1.020 | 0.310 |
| Census tract restrnt sprt per sq.mi. | 0.002 | 1.002 | 0.003 | 0.570 | 0.570 |
| County population | -0.000 | 1.000 | 0.000 | -1.970 | 0.049 |
| County viol. crime per 100k | 0.000 | 1.000 | 0.000 | 1.000 | 0.320 |
| County property crime per 100k | -0.000 | 1.000 | 0.000 | -1.090 | 0.280 |
| County firearm suicide / suicide | -0.806 | 0.447 | 0.385 | -2.090 | 0.037 |

Table A6.16. Cox proportional hazards model coefficients for the model with subsequent arrest for a any violent crime as the outcome and prior arrest for aggravated assault as the primary exposure.

| Variable | coef | exp(coef) | se(coef) | z | p |
| --- | --- | --- | --- | --- | --- |
| Non-vehi. violation only | 1.332 | 3.787 | 0.036 | 36.85 | 0.000 |
| Vehi. violation only | 1.367 | 3.924 | 0.169 | 8.090 | 0.000 |
| Vehi. violation and non-vehi. violation | 1.813 | 6.130 | 0.056 | 32.59 | 0.000 |
| Male | 0.379 | 1.461 | 0.074 | 5.120 | 0.000 |
| Age at index purchase | -0.043 | 0.958 | 0.002 | -19.78 | 0.000 |
| Num. guns purchased prior to index | -0.008 | 0.992 | 0.004 | -2.170 | 0.030 |
| Census tract population | -0.000 | 1.000 | 0.000 | -0.800 | 0.430 |
| Census tract prop. male | 0.004 | 1.004 | 0.006 | 0.660 | 0.510 |
| Census tract SES index | -0.214 | 0.807 | 0.025 | -8.540 | 0.000 |
| Census tract pop. per sq.mi. | 0.000 | 1.000 | 0.000 | 1.290 | 0.200 |
| Census tract prop. age 20-24 of 20-44 | -0.003 | 0.997 | 0.004 | -0.700 | 0.490 |
| Census tract bars/pubs per sq.mi. | -0.003 | 0.997 | 0.005 | -0.580 | 0.560 |
| Census tract off-premise per sq.mi. | 0.004 | 1.004 | 0.003 | 1.190 | 0.230 |
| Census tract restrnt br/wn per sq.mi. | -0.004 | 0.996 | 0.004 | -0.980 | 0.330 |
| Census tract restrnt sprt per sq.mi. | 0.001 | 1.001 | 0.003 | 0.490 | 0.620 |
| County population | -0.000 | 1.000 | 0.000 | -2.100 | 0.036 |
| County viol. crime per 100k | 0.000 | 1.000 | 0.000 | 1.060 | 0.290 |
| County property crime per 100k | -0.000 | 1.000 | 0.000 | -1.070 | 0.280 |
| County firearm suicide / suicide | -0.751 | 0.472 | 0.378 | -1.990 | 0.047 |

Table A6.17. Cox proportional hazards model coefficients for the model with subsequent arrest for a any violent crime as the outcome and prior arrest for vehicle violation as the primary exposure.

| Variable | coef | exp(coef) | se(coef) | z | p |
| --- | --- | --- | --- | --- | --- |
| Non-weapons only | 1.376 | 3.960 | 0.036 | 38.15 | 0.000 |
| Weapons only | 1.141 | 3.130 | 0.118 | 9.660 | 0.000 |
| Weapons and non-weapons | 1.712 | 5.537 | 0.059 | 29.18 | 0.000 |
| Male | 0.379 | 1.461 | 0.074 | 5.120 | 0.000 |
| Age at index purchase | -0.043 | 0.958 | 0.002 | -19.76 | 0.000 |
| Num. guns purchased prior to index | -0.009 | 0.991 | 0.004 | -2.380 | 0.017 |
| Census tract population | -0.000 | 1.000 | 0.000 | -0.870 | 0.380 |
| Census tract prop. male | 0.004 | 1.004 | 0.006 | 0.650 | 0.520 |
| Census tract SES index | -0.211 | 0.810 | 0.025 | -8.410 | 0.000 |
| Census tract pop. per sq.mi. | 0.000 | 1.000 | 0.000 | 1.410 | 0.160 |
| Census tract prop. age 20-24 of 20-44 | -0.002 | 0.998 | 0.004 | -0.590 | 0.550 |
| Census tract bars/pubs per sq.mi. | -0.003 | 0.997 | 0.005 | -0.600 | 0.550 |
| Census tract off-premise per sq.mi. | 0.003 | 1.003 | 0.003 | 1.130 | 0.260 |
| Census tract restrnt br/wn per sq.mi. | -0.005 | 0.995 | 0.004 | -1.100 | 0.270 |
| Census tract restrnt sprt per sq.mi. | 0.002 | 1.002 | 0.003 | 0.630 | 0.530 |
| County population | -0.000 | 1.000 | 0.000 | -2.120 | 0.034 |
| County viol. crime per 100k | 0.000 | 1.000 | 0.000 | 1.140 | 0.260 |
| County property crime per 100k | -0.000 | 1.000 | 0.000 | -1.180 | 0.240 |
| County firearm suicide / suicide | -0.756 | 0.470 | 0.384 | -1.970 | 0.049 |

Table A6.18. Cox proportional hazards model coefficients for the model with subsequent arrest for a any violent crime as the outcome and prior arrest for vehicle violation as the primary exposure.

| Variable | coef | exp(coef) | se(coef) | z | p |
| --- | --- | --- | --- | --- | --- |
| Non-other crimes only | 1.356 | 3.879 | 0.037 | 37.14 | 0.000 |
| Other crimes only | 1.067 | 2.908 | 0.156 | 6.820 | 0.000 |
| Other crimes and non-other crimes | 1.707 | 5.510 | 0.054 | 31.70 | 0.000 |
| Male | 0.387 | 1.473 | 0.074 | 5.230 | 0.000 |
| Age at index purchase | -0.043 | 0.958 | 0.002 | -19.83 | 0.000 |
| Num. guns purchased prior to index | -0.008 | 0.992 | 0.004 | -2.230 | 0.026 |
| Census tract population | -0.000 | 1.000 | 0.000 | -0.790 | 0.430 |
| Census tract prop. male | 0.004 | 1.004 | 0.006 | 0.640 | 0.520 |
| Census tract SES index | -0.214 | 0.807 | 0.025 | -8.540 | 0.000 |
| Census tract pop. per sq.mi. | 0.000 | 1.000 | 0.000 | 1.290 | 0.200 |
| Census tract prop. age 20-24 of 20-44 | -0.003 | 0.997 | 0.004 | -0.650 | 0.520 |
| Census tract bars/pubs per sq.mi. | -0.003 | 0.997 | 0.005 | -0.570 | 0.570 |
| Census tract off-premise per sq.mi. | 0.004 | 1.004 | 0.003 | 1.200 | 0.230 |
| Census tract restrnt br/wn per sq.mi. | -0.005 | 0.995 | 0.004 | -1.090 | 0.280 |
| Census tract restrnt sprt per sq.mi. | 0.002 | 1.002 | 0.003 | 0.580 | 0.560 |
| County population | -0.000 | 1.000 | 0.000 | -2.090 | 0.037 |
| County viol. crime per 100k | 0.000 | 1.000 | 0.000 | 1.160 | 0.250 |
| County property crime per 100k | -0.000 | 1.000 | 0.000 | -1.150 | 0.250 |
| County firearm suicide / suicide | -0.778 | 0.459 | 0.384 | -2.030 | 0.043 |

Table A6.19. Cox proportional hazards model coefficients for the model with subsequent arrest for a any violent crime as the outcome and prior arrest for other crime as the primary exposure.

| Variable | coef | exp(coef) | se(coef) | z | p |
| --- | --- | --- | --- | --- | --- |
| Non-theft only | 1.371 | 3.937 | 0.036 | 38.04 | 0.000 |
| Theft only | 1.092 | 2.982 | 0.121 | 9.020 | 0.000 |
| Theft and non-theft | 1.772 | 5.883 | 0.059 | 29.82 | 0.000 |
| Male | 0.392 | 1.479 | 0.074 | 5.280 | 0.000 |
| Age at index purchase | -0.043 | 0.958 | 0.002 | -19.77 | 0.000 |
| Num. guns purchased prior to index | -0.008 | 0.992 | 0.004 | -2.230 | 0.026 |
| Census tract population | -0.000 | 1.000 | 0.000 | -0.880 | 0.380 |
| Census tract prop. male | 0.004 | 1.004 | 0.006 | 0.640 | 0.520 |
| Census tract SES index | -0.215 | 0.807 | 0.025 | -8.580 | 0.000 |
| Census tract pop. per sq.mi. | 0.000 | 1.000 | 0.000 | 1.470 | 0.140 |
| Census tract prop. age 20-24 of 20-44 | -0.003 | 0.997 | 0.004 | -0.680 | 0.500 |
| Census tract bars/pubs per sq.mi. | -0.004 | 0.996 | 0.005 | -0.690 | 0.490 |
| Census tract off-premise per sq.mi. | 0.003 | 1.003 | 0.003 | 1.050 | 0.290 |
| Census tract restrnt br/wn per sq.mi. | -0.005 | 0.995 | 0.004 | -1.030 | 0.300 |
| Census tract restrnt sprt per sq.mi. | 0.002 | 1.002 | 0.003 | 0.590 | 0.560 |
| County population | -0.000 | 1.000 | 0.000 | -2.150 | 0.031 |
| County viol. crime per 100k | 0.000 | 1.000 | 0.000 | 1.140 | 0.250 |
| County property crime per 100k | -0.000 | 1.000 | 0.000 | -1.170 | 0.240 |
| County firearm suicide / suicide | -0.756 | 0.469 | 0.383 | -1.980 | 0.048 |

Table A6.20. Cox proportional hazards model coefficients for the model with subsequent arrest for a any violent crime as the outcome and prior arrest for theft as the primary exposure.

| Variable | coef | exp(coef) | se(coef) | z | p |
| --- | --- | --- | --- | --- | --- |
| Non-drug only | 1.377 | 3.965 | 0.036 | 38.73 | 0.000 |
| Drug only | 1.080 | 2.943 | 0.136 | 7.940 | 0.000 |
| Drug and non-drug | 1.767 | 5.854 | 0.063 | 28.18 | 0.000 |
| Male | 0.386 | 1.471 | 0.074 | 5.210 | 0.000 |
| Age at index purchase | -0.043 | 0.957 | 0.002 | -19.95 | 0.000 |
| Num. guns purchased prior to index | -0.008 | 0.992 | 0.004 | -2.150 | 0.031 |
| Census tract population | -0.000 | 1.000 | 0.000 | -0.860 | 0.390 |
| Census tract prop. male | 0.004 | 1.004 | 0.006 | 0.600 | 0.550 |
| Census tract SES index | -0.214 | 0.807 | 0.025 | -8.560 | 0.000 |
| Census tract pop. per sq.mi. | 0.000 | 1.000 | 0.000 | 1.400 | 0.160 |
| Census tract prop. age 20-24 of 20-44 | -0.003 | 0.997 | 0.004 | -0.670 | 0.500 |
| Census tract bars/pubs per sq.mi. | -0.003 | 0.997 | 0.005 | -0.620 | 0.530 |
| Census tract off-premise per sq.mi. | 0.004 | 1.004 | 0.003 | 1.210 | 0.230 |
| Census tract restrnt br/wn per sq.mi. | -0.005 | 0.995 | 0.004 | -1.040 | 0.300 |
| Census tract restrnt sprt per sq.mi. | 0.002 | 1.002 | 0.003 | 0.550 | 0.580 |
| County population | -0.000 | 1.000 | 0.000 | -2.060 | 0.040 |
| County viol. crime per 100k | 0.000 | 1.000 | 0.000 | 1.060 | 0.290 |
| County property crime per 100k | -0.000 | 1.000 | 0.000 | -1.100 | 0.270 |
| County firearm suicide / suicide | -0.784 | 0.457 | 0.384 | -2.040 | 0.041 |

Table A6.21. Cox proportional hazards model coefficients for the model with subsequent arrest for a any violent crime as the outcome and prior arrest for drug abuse crime as the primary exposure.

| Variable | coef | exp(coef) | se(coef) | z | p |
| --- | --- | --- | --- | --- | --- |
| Non-DUI only | 1.378 | 3.969 | 0.036 | 38.74 | 0.000 |
| DUI only | 1.210 | 3.352 | 0.126 | 9.630 | 0.000 |
| DUI and non-DUI | 1.738 | 5.687 | 0.063 | 27.42 | 0.000 |
| Male | 0.383 | 1.467 | 0.074 | 5.180 | 0.000 |
| Age at index purchase | -0.043 | 0.958 | 0.002 | -19.96 | 0.000 |
| Num. guns purchased prior to index | -0.008 | 0.992 | 0.004 | -2.150 | 0.031 |
| Census tract population | -0.000 | 1.000 | 0.000 | -0.830 | 0.410 |
| Census tract prop. male | 0.004 | 1.004 | 0.006 | 0.590 | 0.550 |
| Census tract SES index | -0.214 | 0.807 | 0.025 | -8.570 | 0.000 |
| Census tract pop. per sq.mi. | 0.000 | 1.000 | 0.000 | 1.390 | 0.160 |
| Census tract prop. age 20-24 of 20-44 | -0.003 | 0.997 | 0.004 | -0.690 | 0.490 |
| Census tract bars/pubs per sq.mi. | -0.003 | 0.997 | 0.005 | -0.590 | 0.560 |
| Census tract off-premise per sq.mi. | 0.004 | 1.004 | 0.003 | 1.230 | 0.220 |
| Census tract restrnt br/wn per sq.mi. | -0.005 | 0.995 | 0.004 | -1.030 | 0.300 |
| Census tract restrnt sprt per sq.mi. | 0.001 | 1.001 | 0.003 | 0.510 | 0.610 |
| County population | -0.000 | 1.000 | 0.000 | -2.120 | 0.034 |
| County viol. crime per 100k | 0.000 | 1.000 | 0.000 | 1.120 | 0.260 |
| County property crime per 100k | -0.000 | 1.000 | 0.000 | -1.170 | 0.240 |
| County firearm suicide / suicide | -0.797 | 0.451 | 0.381 | -2.090 | 0.036 |

Table A6.22. Cox proportional hazards model coefficients for the model with subsequent arrest for a any violent crime as the outcome and prior arrest for driving under the influence as the primary exposure.
