# Supplementary material for: Importance of categories of crime for predicting future violent crime among handgun purchasers in California
Source: Inj Epidemiol. 2023 Nov 9;10:57. doi: 10.1186/s40621-023-00462-5 (PMC10634023; doi:10.1186/s40621-023-00462-5)
Supplement: Supplementary file 7 — Additional file 7. CIV conviction sensitivity analysis results table–estimated adjusted hazard ratios and Bonferroni-adjusted family-wise 95% confidence intervals for conviction for a CIV offense. [file 40621_2023_462_MOESM7_ESM.docx]

Additional File 7. Adjusted hazard ratios for conviction for a Crime Index-listed violent crime.

|  | CIV |
| --- | --- |
| Criminal history | Estimate (95% CI) |
| Simple assault |  |
| Only simple assault | 4.1 (2.0, 8.2) |
| Simple assault and other categories | 7.4 (5.7, 9.5) |
| Aggravated assault |  |
| Only aggravated assault | 4.9 (2.5, 9.6) |
| Aggravated assault and other categories | 7.9 (6.0, 10.4) |
| Vehicle |  |
| Only vehicle | 3.7 (1.7, 8.4) |
| Vehicle and other categories | 5.7 (4.4, 7.4) |
| Weapons |  |
| Only weapons | 3.6 (2.1, 6.0) |
| Weapons and other categories | 5.9 (4.5, 7.6) |
| Other crimes (UCR 26) |  |
| Only UCR 26 | 1.2 (0.4, 3.8) |
| UCR 26 and other categories | 5.0 (3.9, 6.5) |
| Theft |  |
| Only theft | 3.2 (1.8, 5.6) |
| Theft and other categories | 5.3 (3.9, 6.5) |
| Drug abuse |  |
| Only drug abuse | 6.2 (3.9, 9.9) |
| Drug abuse and other categories | 6.4 (4.8. 8.5) |
| DUI |  |
| Only DUI | 2.4 (1.2, 4.9) |
| DUI and other categories | 6.3 (4.7, 8.4) |

Table A7.1. Adjusted hazard ratios for time to conviction with corresponding 95% family-wise confidence intervals for crime categories with high relative influence.


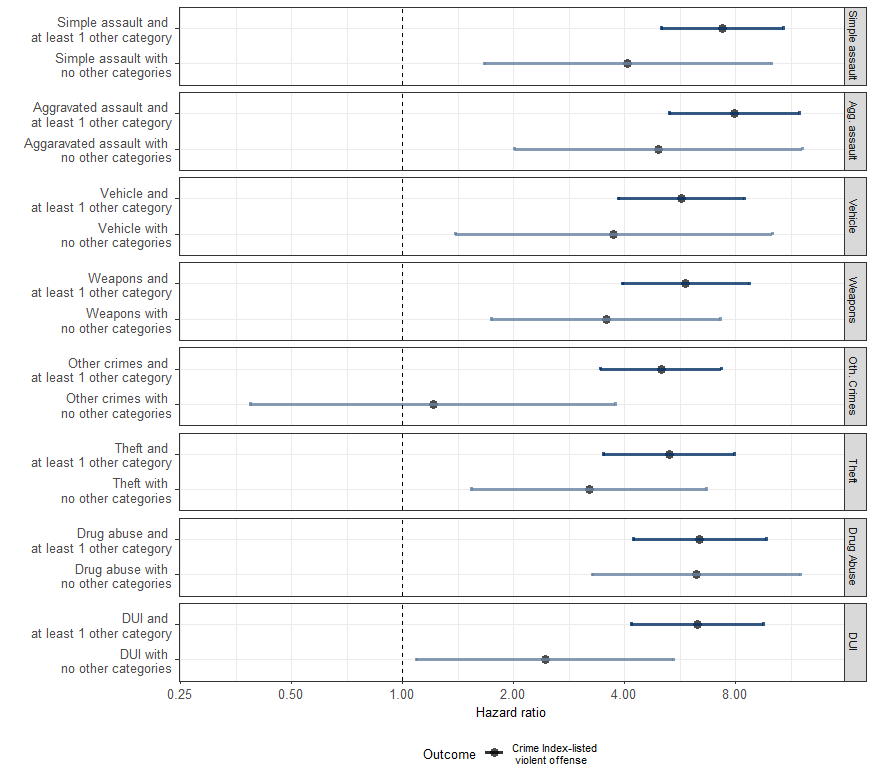


Figure A7.1. Adjusted hazard ratios and corresponding 95% family-wise confidence intervals by isolated UCR categories and multiple UCR categories.
