# Supplementary material for: Importance of categories of crime for predicting future violent crime among handgun purchasers in California
Source: Inj Epidemiol. 2023 Nov 9;10:57. doi: 10.1186/s40621-023-00462-5 (PMC10634023; doi:10.1186/s40621-023-00462-5)
Supplement: Supplementary file 8 — Additional file 8. Unadjusted model sensitivity analysis results table–estimated unadjusted hazard ratios and corresponding family-wise 95% confidence interval values for all outcomes and exposures. [file 40621_2023_462_MOESM8_ESM.docx]

Additional File 8. Unadjusted hazard ratios for arrest for a violent crime.

|  | CIV | Firearm violence | Any violence |
| --- | --- | --- | --- |
| Criminal history | Estimate (95% CI) | Estimate (95% CI) | Estimate (95% CI) |
| Simple assault |  |  |  |
| Only simple assault | 4.2 (2.9, 6.2) | 4.9 (2.5, 9.5) | 3.8 (2.8, 5.1) |
| Simple assault and other categories | 7.0 (6.1, 8.0) | 5.7 (4.3, 7.5) | 6.8 (6.1, 7.6) |
| Aggravated assault |  |  |  |
| Only aggravated assault | 3.2 (2.0, 5.0) | 3.5 (1.6, 8.0) | 3.9 (2.8, 5.3) |
| Aggravated assault and other categories | 7.5 (6.5, 8.7) | 6.7 (5.0, 8.9) | 6.7 (5.9, 7.5) |
| Vehicle |  |  |  |
| Only vehicle | 3.1 (1.9, 5.2) | 3.0 (1.1, 8.0) | 4.6 (3.3, 6.4) |
| Vehicle and other categories | 6.3 (5.4, 7.2) | 5.6 (4.3, 7.4) | 6.5 (5.8, 7.2) |
| Weapons |  |  |  |
| Only weapons | 3.6 (2.7, 4.8) | 3.9 (2.3, 6.6) | 3.3 (2.6, 4.1) |
| Weapons and other categories | 5.8 (5.0, 6.7) | 5.7 (4.3, 7.5) | 5.8 (5.2, 6.5) |
| Other crimes (UCR 26) |  |  |  |
| Only UCR 26 | 3.0 (2.0, 4.5) | -- | 3.2 (2.4, 4.4) |
| UCR 26 and other categories | 5.7 (5.0, 6.7) | -- | 5.7 (5.2, 6.4) |
| Theft |  |  |  |
| Only theft | 2.8 (2.0, 3.9) | 2.2 (1.1, 4.4) | 3.2 (2.5, 4.1) |
| Theft and other categories | 5.9 (5.1, 6.9) | 6.3 (4.8, 8.3) | 6.2 (5.5, 6.9) |
| Drug abuse |  |  |  |
| Only drug abuse | 3.3 (2.4, 4.6) | -- | 3.0 (2.3, 4.0) |
| Drug abuse and other categories | 5.7 (4.8. 6.6) | -- | 5.8 (5.1, 6.5) |
| DUI |  |  |  |
| Only DUI | 3.1 (2.2, 4.4) | 3.2 (1.7, 6.0) | 3.6 (2.8, 4.6) |
| DUI and other categories | 5.6 (4.8, 6.6) | 5.9 (4.4, 7.9) | 5.6 (5.0, 6.3) |

Table A8.1. Unadjusted hazard ratios for time to arrest with corresponding 95% family-wise confidence intervals for crime categories with high relative influence.


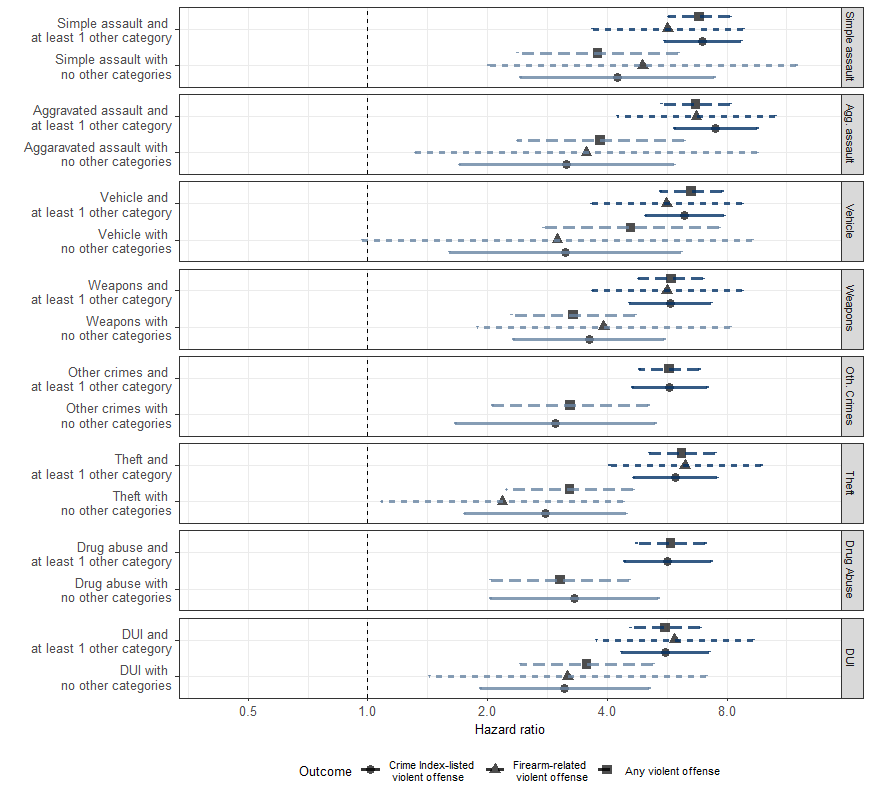


Figure A8.1. Unadjusted hazard ratios and corresponding 95% family-wise confidence intervals by isolated UCR categories and multiple UCR categories.
